# Increased low-molecular-weight mucins in muco-obstructive airway disease limit *Staphylococcus aureus* growth

**DOI:** 10.1128/iai.00693-25

**Published:** 2026-06-04

**Authors:** Caitlyn C. Sebastian, RaNashia Boone, Susan E. Birket, Megan R. Kiedrowski

**Affiliations:** 1 Division of Pulmonary, Allergy and Critical Care, Department of Medicine, Heersink School of Medicine, The University of Alabama at Birmingham 164494, Birmingham, Alabama, USA; University of Illinois Chicago, Chicago, Illinois, USA

**Keywords:** mucin, *Staphylococcus aureus*, aggregation, muco-obstructive airway disease, biofilms

## Abstract

Muco-obstructive airway diseases result in an increase in mucus accumulation and a decrease in mucus clearance. MUC5B is the most abundant secreted mucin in the human airways, and MUC5B mucin strands dimerize to create the mucus mesh network in the healthy respiratory tract. In muco-obstructive airway diseases like cystic fibrosis (CF), immune cells and bacteria release enzymes that degrade MUC5B into smaller fragments that become entangled and compacted, contributing to pathogenesis. We utilized synthetic cystic fibrosis sputum media (SCFM) to examine how mucin polymers can impact *Staphylococcus aureus*, a common CF pathogen that persists despite highly effective modulator therapies to correct CF disease. We found that low-molecular-weight (LMW) mucin negatively impacts *S. aureus* survival and biofilm biomass compared to high-molecular-weight (HMW) mucin. Adding extracellular DNA to SCFM with LMW mucin was not sufficient to restore growth. LMW mucin had a broad negative impact on *S. aureus* laboratory strains and CF clinical isolates. We next tested other CF pathogens, including *Pseudomonas aeruginosa* and nontypeable *Haemophilus influenzae*, and saw no significant differences in growth in HMW or LMW mucin. LMW mucin did not significantly impact *Staphylococcus epidermidis* growth, indicating that there may be specific interactions with *S. aureus*. Overall, this work highlights how interactions with pathogenic mucins may limit *S. aureus* growth in the diseased airways while supporting low-level persistence, and its ability to thrive in the presence of longer mucin strands may help explain why *S. aureus* is well adapted to survive in the healthy respiratory tract.

## INTRODUCTION

Muco-obstructive airway diseases, such as cystic fibrosis (CF), primary ciliary dyskinesia (PCD), and chronic obstructive pulmonary disease (COPD), are characterized by decreased mucociliary clearance and increased mucus accumulation in the airways (1, 2). The respiratory tract mucosal barrier contains heavily glycosylated tethered and secreted mucins that prevent the adhesion of foreign particles and provide scaffolding for antibodies and other antimicrobial materials (3). In muco-obstructive diseases, secreted mucins become compacted and entangled due to hypersecretion and dehydration, which leads to abnormalities in mucin folding (2, 4). Of the secreted mucins, MUC5B is the most abundant in human airways (5). MUC5B is secreted from submucosal glands as linear strands and elongated through dimerization of disulfide linkages, creating the mucus mesh network (6–8). However, in muco-obstructive diseases including CF, mucin degradation is commonly observed due in part to persistent inflammation and infection. Bacteria and immune cells in the diseased airways contribute to mucin degradation through the release of proteases, resulting in an increased abundance of short MUC5B strands and compromising the function of the mucus layer (4, 9, 10). Compacted stagnant mucin creates a unique niche that allows for bacteria to access nutrients resulting from degraded airway proteins and use the compacted mucin mesh as scaffolding for aggregation and biofilm formation (1, 11–13).

*Staphylococcus aureus* is a common respiratory pathobiont that asymptomatically colonizes the upper respiratory tract of approximately 40% of healthy adults (14, 15). High prevalence of *S. aureus* has been reported in people with PCD and CF and is associated with a higher risk of severe disease outcomes (16, 17). More recent studies indicate that the increased prevalence of *S. aureus* leads to higher mortality in people with COPD (18). *S. aureus* is known to bind to mucin strands and can utilize products from degraded mucins broken down by other bacteria in the upper respiratory tract (19, 20). In CF, *S. aureus* remains prevalent in the airways even after treatment with highly effective modulator therapies (HEMT), with continued *S. aureus* colonization observed up to 3 years post-HEMT treatment (21, 22). The use of HEMT potentiates functional cystic fibrosis transmembrane conductance regulator (CFTR) ion channels, which restores airway surface liquid hydration and allows for the clearance of the mucus out of the airways (21, 23). Together with microbiological data, this suggests *S. aureus* is adapted to persist in both the diseased airways in the presence of high mucin concentrations and in airways with restored mucociliary clearance. However, how interactions with mucins impacts *S. aureus* biogeography and persistence in the mucus-obstructed airways has not been explored in great depth.

Synthetic cystic fibrosis sputum media (SCFM) mimics the nutritional environment of CF and can include extracellular DNA (eDNA) and mucin polymers that can be added in different concentrations to replicate the increased eDNA and mucin concentrations found in muco-obstructive airway diseases (1, 12, 24, 25). Bacterial transcriptomes in SCFM have been shown to be comparable to *ex vivo* CF sputum collected from persons with CF (pwCF) and other commonly used CF models (26, 27). While there are currently no widely available synthetic defined medium models for other muco-obstructive airway diseases, there is evidence of similar changes in metabolic profiles in the respiratory tract, such as an increase in amino acids, lactate, phospholipids, and sialic acids, in people with muco-obstructive airway disease compared to healthy individuals (28, 29).

Our work aimed to investigate how *S. aureus* interactions with mucins affect persistence in the CF airway environment. In this study, we used SCFM with different mucin polymer sizes to determine how smaller mucin strands impact *S. aureus* compared to longer strands, mimicking degraded pathogenic mucins found in disease and healthy airway mucins. We found that shorter mucin strands had a negative impact on *S. aureus* survival and biomass compared to longer mucin polymers for laboratory strains and clinical isolates, and reducing the concentration of smaller mucins partially recovered *S. aureus* survival and biofilm. Additionally, we did not see a negative effect of small mucin polymers on other CF pathogens tested, including *Pseudomonas aeruginosa* or nontypeable *Haemophilus influenzae* (NTHi). When we evaluated the survival of another *Staphylococcus* species, *Staphylococcus epidermidis,* we did not see a decrease in survival in the presence of small mucin polymers, indicating that this phenotype may be specific to *S. aureus*.

## RESULTS

### Low-molecular-weight mucin limits *S. aureus* growth and reduces biofilm biomass

**Fig 1:**
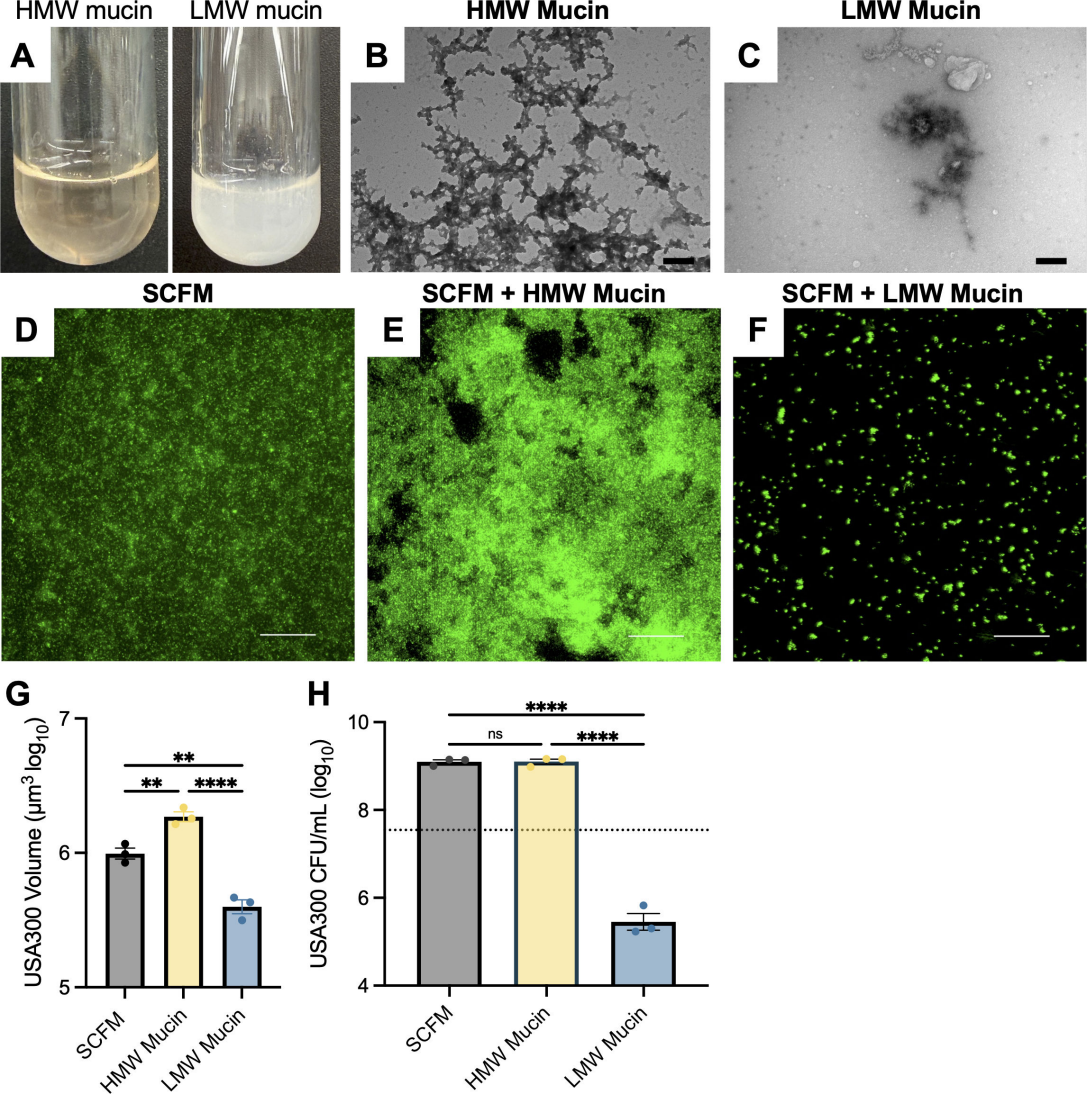
Low-molecular-weight mucins reduce *S. aureus* USA300 survival and biomass. Images of HMW mucin (left) and LMW mucin (right) after being dissolved in SCFM (**A**). Transmission electron microscopy of uninfected (**B**) HMW mucin or (**C**) LMW mucin. Representative images of GFP-expressing USA300 (**D**) in SCFM, SCFM + HMW mucin (**E**), or SCFM + LMW mucin (**F**) z-stack images taken at 24 h. Quantification of biomass (**G**) is represented by averaging five fields of view from three biological replicates for each condition. USA300 growth (**H**) in SCFM (gray), HMW mucin (yellow), or LMW mucin (blue) for 24 h, shaking. The dotted line represents the average inoculum under the conditions. Scale bar represents 50 µm in z-Stack images. Scale bars in TEM images represent 200 nm. One-way ANOVA was performed on USA300 colony-forming units (CFUs) on average biomass. (**P < 0.01, ***P < 0.001, ****P < 0.0001, ns = nonsignificant). Data are represented by mean ± SEM.

To determine the impact of long and short mucin polymers, we utilized two different commercially available bovine submaxillary mucin (BSM) products of different molecular masses to represent a low-molecular-weight (LMW; 200–500 kDa) and high-molecular-weight (HMW; 400–4,000 kDa) mucin environment in CF. Once solubilized in SCFM, the mucins were clearly distinguishable from one another, with LMW mucin having a cloudy appearance in solution (Fig. 1A). Transmission electron microscopy confirmed longer mucin polymers were present in the HMW mucin samples, with the LMW samples forming dense aggregates (Fig. 1B and C; Fig. S1). We inoculated a well-characterized, community-associated strain of methicillin-resistant *S. aureus* (MRSA) USA300 into SCFM without polymers and SCFM containing LMW or HMW mucin. Using GFP-expressing USA300, we examined how LMW and HMW mucins impact USA300 biofilm formation. We found a significant decrease in overall biomass in the LMW mucin condition compared to the HMW mucin and no polymer control conditions (Fig. 1D through G). We also observed that there was no significant difference in *S. aureus* colony-forming units (CFU) after 24 h of incubation in SCFM alone or SCFM with HMW mucin; however, there was a significant decrease in USA300 survival in SCFM with LMW mucin (Fig. 1H).

To determine how other mucin sources affect *S. aureus* growth, we inoculated USA300 in porcine gastric mucin (PGM), a more readily available, cheaper alternative to BSM that has been previously used to replicate a CF environment (9, 27). We found that USA300 was able to survive and grow in PGM (size range of 4,000–5,500 kDa) to a similar level observed in SCFM without polymers and SCFM with HMW bovine salivary mucin (Fig. S2). Other groups have previously analyzed the same LMW BSM product we use in these studies and found this mucin to be >98% pure, with the only other content reported to be bovine serum albumin, which we would not expect to have anti-bacterial effects (30, 31). However, to ensure that there were no contaminating small molecules present in the LMW mucin sample that could be conferring antimicrobial effects, we centrifuged SCFM containing LMW mucin through a 100 kDa cutoff centrifugal filter and collected the filtrate to perform growth assays (Fig. S3A). We found that USA300 did not have a growth defect in the <100 kDa filtrate, and the total CFU after 24 h resembled the SCFM without mucin control (Fig. S3B). Together, these results indicate that LMW mucin polymers have a detrimental effect on USA300.

**Fig 2:**
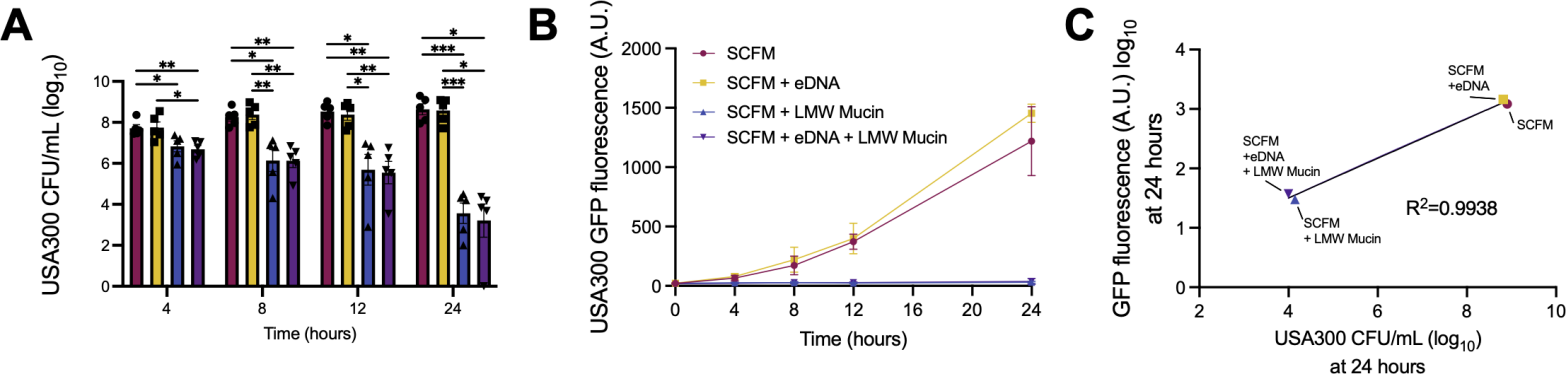
Low-molecular-weight mucins limit *S. aureus* USA300 survival over time. USA300 growth (**A**) in SCFM (red), SCFM + eDNA (yellow), SCFM + LMW Mucin (blue), and SCFM + eDNA + LMW Mucin (purple) at 37°C for 4, 8, 12, and 24 h. USA300 labeled with green fluorescent protein (GFP) fluorescence (**B**) was measured at 4, 8, 12, and 24 h in a plate reader. Correlation plot (**C**) of USA300 CFUs and GFP fluorescence at 24 h (R^2^ = 0.9938). Two-way ANOVA was performed on USA300 CFUs. (*P ≤ 0.05, **P < 0.01 ***P < 0.001, ns = nonsignificant.) Data are represented by mean ± SEM.

To further determine how LMW mucins impact USA300 growth over time, we inoculated USA300 into SCFM containing LMW mucin and measured CFUs at 4, 8, 12, and 24 h post-inoculation. We also inoculated USA300 into SCFM containing eDNA, both with and without LMW mucin, to determine if eDNA could rescue USA300. At 4 h, we already saw a significant decrease in USA300 CFUs in SCFM with LMW mucin and SCFM with LMW mucin and eDNA compared to conditions where LMW mucins were not present (Fig. 2A). We continued to see this significant decrease at later 8, 12, and 24 h time points. There remained no significant difference between SCFM with LMW mucin and SCFM with both LMW mucin and eDNA, indicating that eDNA is not sufficient to rescue USA300 growth in the presence of LMW mucin.

Since the medium containing LMW mucin is opaque, making it difficult to perform a standard growth curve based on optical density at 600 nm, we utilized our GFP-labeled USA300 strain and observed fluorescence intensity over time as a proxy for growth. We measured increased GFP fluorescence over time for USA300 cultured in conditions where LMW mucin was not present, while fluorescence in the media containing LMW mucin remained stagnant (Fig. 2B). We confirmed through a correlation plot utilizing CFUs from the 24 h time point that levels of GFP fluorescence positively correlated with higher CFU counts (R^2^ = 0.9938; Fig. 2C). To confirm the effects of LMW mucins on another strain of MRSA, we repeated these experiments using a USA100 hospital-acquired MRSA strain. We observed that USA100 also had a growth defect in the presence of LMW mucin compared to HMW mucin (Fig. S4A). Additionally, LMW mucin caused a significant decrease in CFUs of USA100 as early as 4 h. At 24 h, LMW mucin decreased GFP fluorescence in mucin-containing conditions (Fig. S4B through D). These results indicate that LWM mucin has a negative growth impact on community and hospital-acquired *S. aureus* MRSA strains.

**Fig 3:**
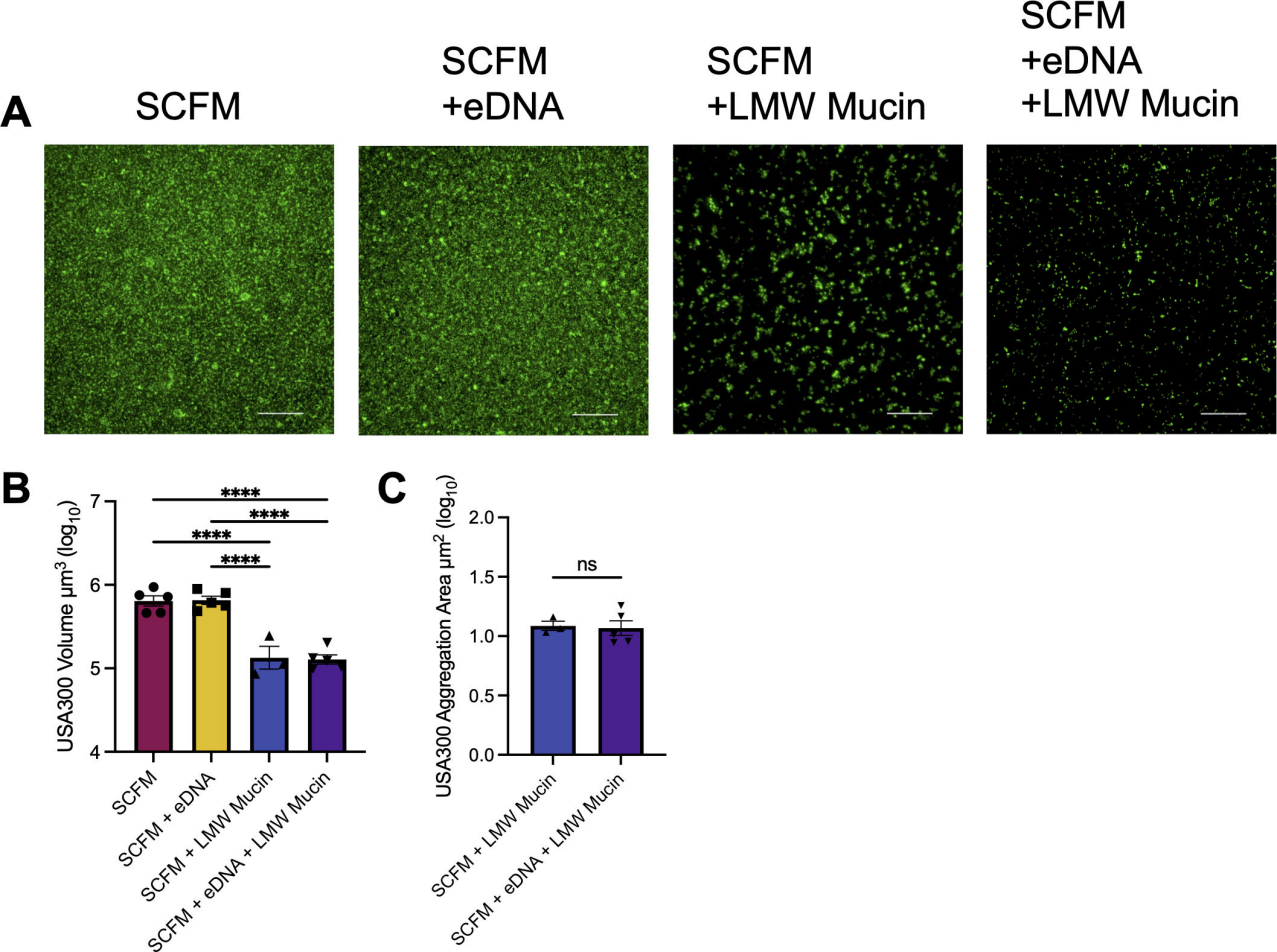
*S. aureus* USA300 biofilm biomass is reduced in the presence of low-molecular-weight mucin. Representative Z-stack images of USA300 (**A**) biofilm in SCFM with and without eDNA or LMW mucins after 24 h. USA300 biomass (**B**) in SCFM with and without eDNA or LMW mucin. Average aggregate size (**C**) of USA300 in SCFM with LMW mucin and with or without eDNA. Biomass and aggregate size are represented by 3–5 biological replicates and five images taken of each condition. One-way ANOVA was performed on volumetric measurement (****P ≤ 0.0001) and Welch’s *t*-test performed on aggregation area (ns = nonsignificant). Data are represented by mean ± SEM.

Although we saw that eDNA could not recover *S. aureus* survival in the presence of LMW mucins, we wanted to confirm if the addition of eDNA changed other aspects of *S. aureus* biofilms, such as overall biomass or aggregate size. Microscopy of GFP-expressing USA300 showed no statistical difference in biomass of biofilms grown in SCFM without polymers or SCFM with eDNA, and *S. aureus* formed confluent biofilms in both conditions (Fig. 3A and B). As expected, LMW mucin significantly decreased biomass volume in media with and without eDNA (Fig. 3A and B). Additionally, the average aggregate area was not found to be affected by the presence of both eDNA and LMW mucin compared to LMW mucin alone (Fig. 3C). We repeated this assay using our GFP-expressing USA100 strain and saw similar results (Fig. S5).

### Reducing LMW mucin concentration partially recovers *S. aureus* growth and increases biomass

**Fig 4:**
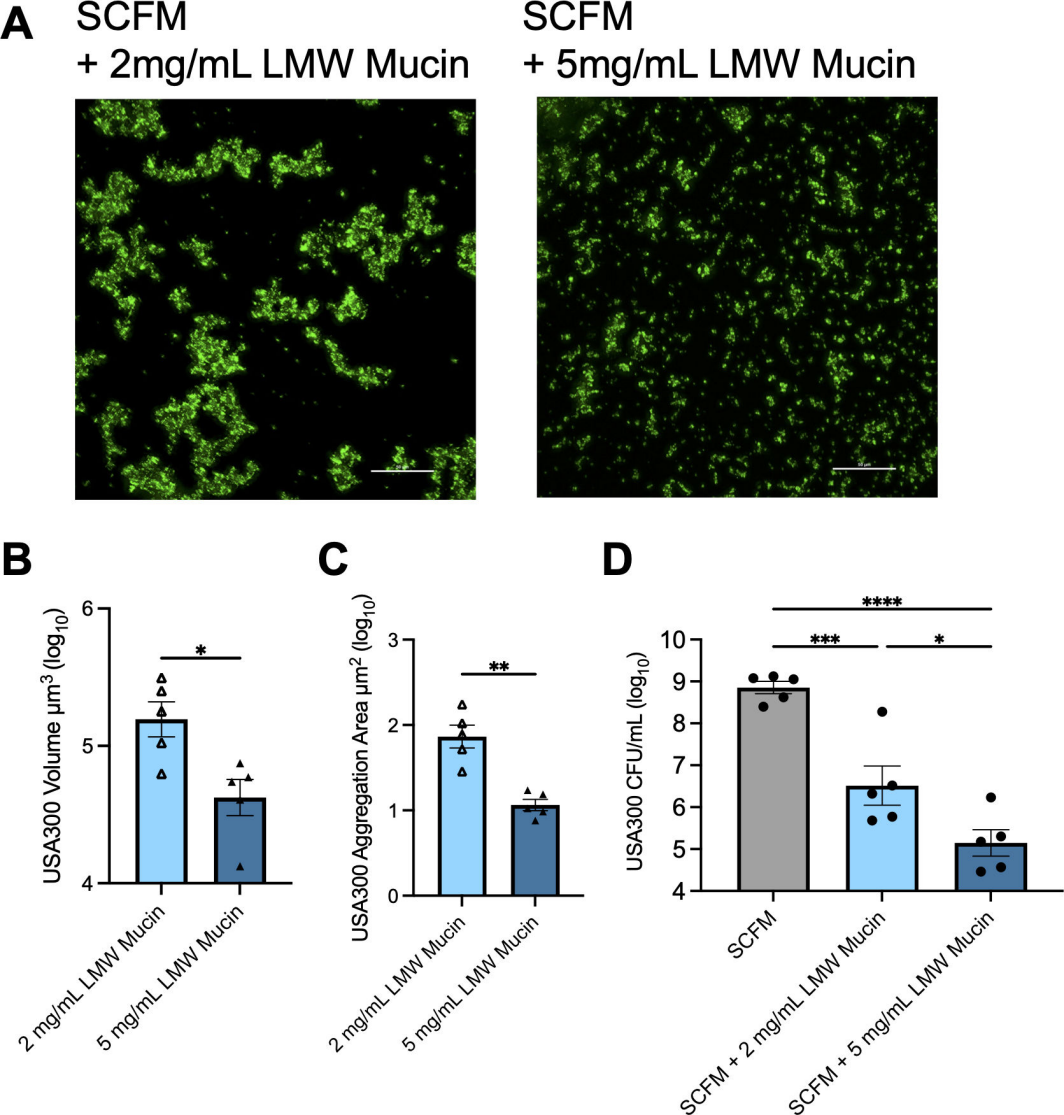
Reduced mucin concentration increases biomass and aggregate size and partially restores *S. aureus* USA300 survival. Representative z-Stack images of USA300 (**A**) in SCFM with either 2 mg/mL mucin (Left) or 5 mg/mL mucin (Right) after 24 h at 40×. Scale bar represents 50 µm. Quantification of (**B**) volumetric and (**C**) aggregate size of 5 biological replications with 3–6 images taken of each well. CFUs of USA300 (**D**) after 24 h of growth. One-way ANOVAs were performed on CFUs, and Welch’s *t*-tests were performed on volume and aggregate area (*P ≤ 0.05,**P ≤ 0.01, ***P ≤ 0.001, ****P ≤ 0.0001). Data are represented by mean ± SEM.

Next, we wanted to determine if reducing the amount of LMW mucin would recover USA300 growth and biofilm biomass. We inoculated GFP USA300 into SCFM with LMW mucin at either 5 mg/mL or 2 mg/mL, representing mucin concentrations in CF or healthy airways, respectively (1), for 24 h. Our images showed larger aggregates in 2 mg/mL LMW mucin compared to the higher 5 mg/mL condition, representative of CF (Fig. 4A). Through quantification of images, we confirmed that there was a significant increase in both biomass and aggregate size in 2 mg/mL LMW mucin compared to 5 mg/mL LMW mucin (Fig. 4B and C). Next, we plated to measure CFUs and found that incubation in 2 mg/mL of LMW mucin resulted in a significant increase in USA300 survival compared to 5 mg/mL of LMW mucin (Fig. 4D). However, there was still a significant decrease overall in USA300 survival compared to SCFM with no mucin. We repeated these experiments with USA100 and saw that USA100 also had a significant increase in biomass, aggregate size, and survival in 2 mg/mL LMW mucin compared to the 5 mg/mL (Fig. S6). Together, these data indicate that reducing the amount of LMW mucin to concentrations found in healthy individuals improves *S. aureus* survival, biomass, and aggregate size.

### LMW mucin broadly impedes the growth of common *S. aureus* laboratory strains and clinical isolates

**Fig 5:**
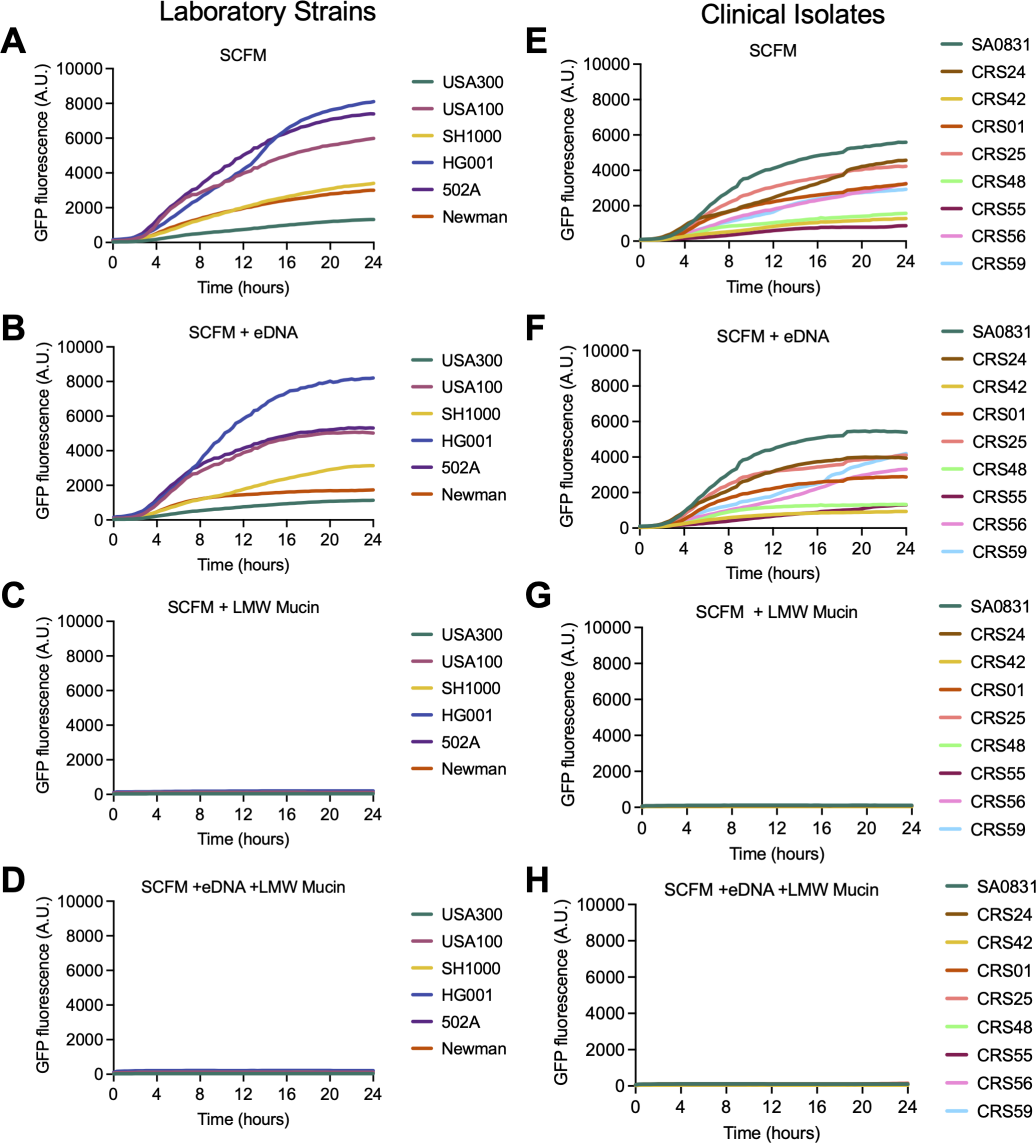
Mucin limits growth of *S. aureus* laboratory strains and clinical isolates in SCFM. Laboratory isolates (**A–D**) and clinical isolates (**E–H**) of *Staphylococcus aureus* were grown in SCFM conditions with GFP fluorescence reads every 20 min for a total of 24 h. Lines represent a mean of 5 biological replicates.

Since we observed that LMW mucin results in a decrease in growth in two well-characterized MRSA strains, we next examined if this phenotype is broadly conserved. We evaluated additional *S. aureus* laboratory strains and *S. aureus* CF clinical isolates, including eight collected from the sinuses of individuals with CF and chronic rhinosinusitis (CRS) and one from the lung (Table S1). We utilized the fluorescence-based growth assay described above in Fig. 2 to screen *S. aureus* strains transduced with the GFP-expression plasmid pCM29 over a 24 h period. First, we determined the level of SCFM and mucin autofluorescence to ensure that all isolates were above the baseline level of autofluorescence. We measured all uninfected conditions and found that there was low autofluorescence that would not interfere with fluorescence resulting from our bacterial reads (Fig. S7). In SCFM without polymers, all laboratory strains and clinical isolates evaluated had an increase in fluorescence over time, but varied in maximum fluorescence observed between strains (Fig. 5A and E; Fig. S8). The addition of eDNA to SCFM yielded similar results to SCFM without polymers (Fig. 5B and F; Fig. S8). As before, we saw that none of the laboratory strains or clinical isolates we tested had an increase in fluorescence over time in LMW mucin but did measure above our baseline levels of autofluorescence (Fig. 5C and G; Fig. S8). We continued to see this trend in SCFM with LMW mucin and eDNA for laboratory strains and clinical isolates (Fig. 5D and H; Fig. S8). Of the isolates tested, only two did not show a significant difference in final fluorescence between control SCFM and mucin-containing conditions at 24 h (laboratory strain Newman and clinical isolate CRS59) (Fig. S8). Together, these data indicate that LMW has a broad negative impact across multiple laboratory and CF clinical isolates.

To determine if these strains can grow in the presence of LMW mucin in a different base growth medium, we dissolved 5 mg/mL of LMW mucin in tryptic soy broth (TSB). We first determined the baseline autofluorescence of uninfected TSB with and without LMW mucin (Fig. S9A). We then infected TSB or TSB with LMW mucin with the same panel of laboratory strains and clinical isolates as used in Fig. 5. Like SCFM without polymers, laboratory strains and clinical isolates exponentially grew in TSB (Fig. S9B and D); however, no strains tested were able to grow in TSB with LMW mucins (Fig. S9C and E). This indicates that LMW mucins are mediating the observed decrease in growth and not the CF nutritional environment represented by SCFM.

### Investigating the effects of LMW mucin on other CF pathogens and respiratory tract colonizing bacteria

**Fig 6:**
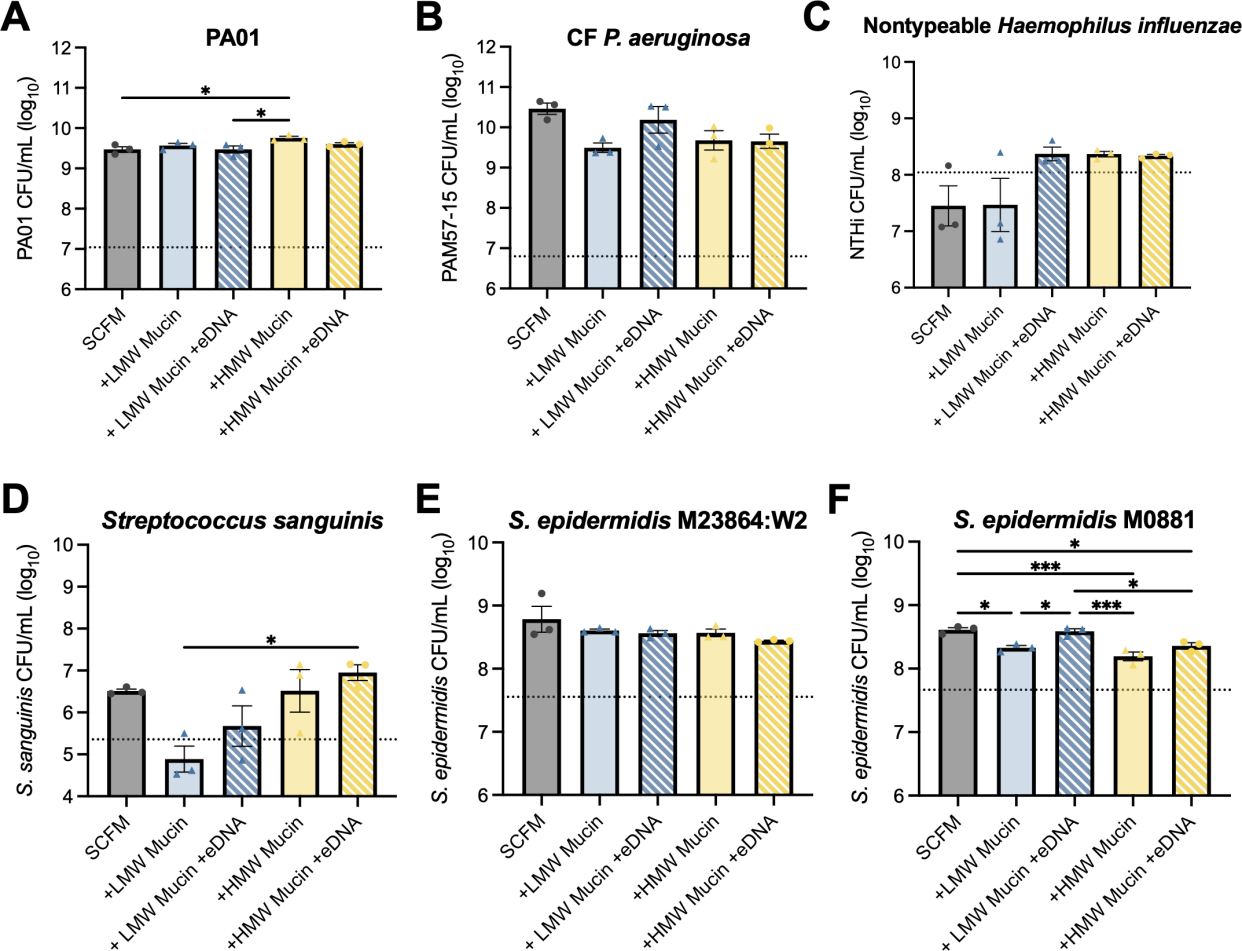
Other CF pathogens and *S. epidermidis* are not negatively impacted by low molecular weight mucin. Growth of PAO1 (**A**), PAM57-15 (**B**), NTHi (**C**), *S. sanguinis* (**D**), *S. epidermidis* M23864:W2 (**E**), and *S. epidermidis* M0881 (**F**) in SCFM (gray), SCFM with LMW (blue), or HMW (yellow) with (striped) or without (solid) eDNA at 24 h. One-way ANOVAs were performed on CFUs (*P ≤ 0.05, **P ≤ 0.01, ***P ≤ 0.001). Data are represented by mean ± SEM.

Since we found that LMW mucin broadly impacts *S. aureus* strains, we next wanted to determine if other CF pathogens are similarly negatively impacted by LMW mucins. *P. aeruginosa* is a common opportunistic pathogen in the CF respiratory tract, and mucoid variants of *P. aeruginosa* are associated with decreased lung function and quality of life in people with CF (32, 33). To test the effects of LMW and HMW mucins on *P. aeruginosa,* we utilized the non-mucoid and mucoid strains PAO1 and PAM57-15, respectively, inoculated into SCFM containing LMW or HMW mucin with or without eDNA. Both PAO1 and PAM57-15 grew approximately 2-logs above the inoculum in all conditions, regardless of the presence of LMW mucin or HMW mucin (Fig. 6A and B). PAO1 growth was significantly increased in SCFM containing HMW mucin alone compared to SCFM without polymers and in SCFM with LMW mucin and eDNA (Fig. 6A). There was no statistical difference between conditions for PAM57-15 (Fig. 6B). Next, we tested another gram-negative CF bacterial pathogen, nontypeable *Haemophilus influenzae* (NTHi). Compared to *P. aeruginosa,* NTHi maintained survival at the level of inoculum with no significant difference between LMW or HMW mucins with or without eDNA (Fig. 6C).

We next asked if other bacteria found in mucosal environments, such as the oral cavity, are impacted by mucins. *Streptococcus sanguinis* is an oral commensal species, and prior studies have shown that increased abundance of some *Streptococcus* species is associated with promoting stable lung function in pwCF (34, 35). While there was no significant difference in growth between SCFM without polymers and SCFM with LMW mucin, we observed a significant increase in *S. sanguinis* survival when grown in SCFM with HMW mucin and eDNA compared to SCFM with only LMW mucin (Fig. 6D). To test if other *Staphylococcus* species are impacted by LMW or HMW mucins, we grew two strains of *S. epidermidis* in the same conditions as above (Fig. 6E and F). There were no significant differences between conditions with *S. epidermidis* M23864:W2 (Fig. 6E). However, *S. epidermidis* M0881 had a significant decrease in growth in SCFM with LMW or HMW mucin compared to SCFM without polymers (Fig. 6F). Additionally, there was a significant increase in CFUs when eDNA was added to the LMW mucin condition, but not the HMW mucin condition (Fig. 6F). Despite these differences, both strains of *S. epidermidis* were able to grow at least a log above the inoculum, indicating that *S. epidermidis* is not experiencing the same negative effects of LMW mucin as *S. aureus* (Fig. 1, 6E and F). Together, these results indicate that the growth-inhibiting phenotype of LMW mucin may be specific to *S. aureus* and not to other species of bacteria that commonly inhabit the CF airways.

## DISCUSSION

Despite the introduction of HEMT to treat CF disease and restore mucociliary clearance, *S. aureus* in chronic bacterial respiratory infections is most often not eradicated (21, 36, 37). In the work presented here, we show that LMW mucins suppress *S. aureus* growth and reduce survival (Fig. 1 and 2). Mucins originating from different regions of the airway mucosa have been described as having varied biophysical properties. Superficial mucus sampled from the surface of bronchial epithelial cells was found to have enhanced solubility compared to mucins produced by submucosal glands (38). TEM images of LMW bovine submaxillary mucins with anti-*S*. *aureus* activity (Fig. 1; Fig. S1) and the opacity of these mucins in solution suggest that these mucins share some resemblance with insoluble mucin particles previously observed by other groups (39). Insoluble mucus strands and mucus flakes have been observed in samples from people with CF as well as CF animal models. Unlike LMW mucins, the HMW mucin polymers we evaluated were able to form structures more closely resembling mucin mesh networks (Fig. 1; Fig. S1). We predict that the inability of LMW polymers to form a mesh network affects interactions of these mucins with other particles in the airway, including bacteria. Previous analysis of the same LMW mucin source we use here in two separate studies found that it contains several major and trace O-glycans (40), as well as multiple N-glycans (30). O-glycans in gastric mucin were previously shown to be important for interactions with *Salmonella* species (41), and it is possible that O-glycans may similarly be responsible for controlling *S. aureus* growth via interactions with secreted airway mucins. The inability of LMW mucin polymers to form a proper mesh network may result in increased availability of glycan side chains to interact with airway bacteria. Future studies could investigate the role of O-glycans in mediating anti-*S*. *aureus* activity through the mutation of O-glycosylation sites or targeted enzymatic treatment.

In CF, increased levels of proteases released from innate immune cells and bacterial pathogens degrade intact airway mucins into smaller polymers (9, 10, 19, 25). These small mucin polymers can alter mucus biochemical properties by changing the elasticity and viscosity of the mucus layer, resulting in decreased mucociliary clearance (25, 42). While there are some reported instances of bacterial degradation of mucus releasing secondary metabolites beneficial to *S. aureus* growth and persistence (19), our results indicate that without mucin-degrading bacteria present, LMW mucin has an inhibitory effect on *S. aureus*. Other studies that have inoculated *S. aureus* into SCFM2 have shown that genes related to iron acquisition are downregulated compared to human sputum samples, while genes related to virulence factors and metabolism are more closely comparable to human sputum samples (12, 43). However, these studies did not specify the mucin polymer size used, and differential gene regulation may occur in the presence of LMW or HMW mucins. Further studies of transcriptional changes in low- and high-molecular-weight mucin would be needed to understand how mucin polymers influence *S. aureus* gene expression.

*S. aureus* biofilm formation is largely governed by the accessory gene regulator (agr) system through the sensing of autoinducing peptides and production of extracellular dispersal molecules, such as nucleases, proteases, and phenol-soluble modulins (PSMs) (44). It has been found that eDNA released from cell lysis is important for *S. aureus* biofilm maturation and attachment (45–47). Our results also indicate that biopolymers in sputum, such as eDNA, are not sufficient to rescue *S. aureus* from the negative effects of LMW mucins. In the experiments presented here, eDNA also did not provide a benefit when *S. aureus* was cultured in HMW mucin. Interestingly, agr mutant *S. aureus* isolates are frequently identified from the respiratory tract, despite the known requirement of a functional agr system to produce secreted virulence factors and toxins associated with acute airway infection (48, 49). Further study of agr-regulated gene expression in the presence of LMW mucins, or potential defects in expression of other biofilm-related genes, would be needed to determine if agr is dysregulated in the presence of LMW mucins. Alternatively, the negative impacts of LMW mucin on *S. aureus* may be dominant to any beneficial effects of eDNA on *S. aureus* biofilms.

Another group determined that *S. aureus* lacking the transcriptional regulator MgrA has a decreased binding affinity to both pig gastric mucin and BSM, but MgrA does not have the same affinity to other biopolymers (50). MgrA is a global regulator known to play a major role in biofilm formation, clumping, and repression of *S. aureus* cell wall-associated proteins (51, 52). MgrA is activated by the two-component system ArlRS, which regulates genes involved in agglutination and other virulence factors (51, 53). There is evidence that the *agr* system can control expression of *mgrA* through mRNA stabilization provided by the regulatory RNA, RNAIII (54, 55). *S. aureus* can also exhibit mucin-induced dispersal through production of PSMs, which are responsible for *S. aureus* biofilm dispersal as well as dispersal of other bacteria, including commensal Corynebacterium species, as members of our group recently demonstrated (56, 57). It is possible that a combination of ArlRS, MgrA, and agr-dependent signaling is needed to mediate survival in the presence of mucin through the production of PSMs and the expression of specific surface-binding proteins.

Different muco-obstructive airway diseases can result in varying levels of mucin concentrations in the respiratory tract. The standard concentration of mucin in SCFM2 is 5 mg/mL, meant to replicate mucin concentrations observed in CF sputum from pwCF (1, 12, 24). Based on the literature, we wanted to replicate the average mucin concentration seen in the healthy population, which averages around 2 mg/mL, for comparison to CF conditions (1, 25). HEMT corrects ion transport across the epithelial layer in the CF airways, and while this has been found to result in restored mucociliary clearance, bacterial pathogens are often not eradicated. The continued persistence of bacteria such as *P. aeruginosa* in the HEMT-modified CF airway environment could result in continued degradation of mucins despite HEMT-mediated correction, leading to the continued presence of pathogenic mucin polymers. Utilizing the LMW mucin at 2 mg/mL, we were able to show that as mucin concentrations decrease, *S. aureus* growth is partially restored, suggesting that *S. aureus* could persist in this environment. Additionally, we show that *S. aureus* growth is not inhibited by HMW mucins, representing longer mucin strands that may be present in the HEMT corrected airway. Taken together, this provides some insight into why we see some bacteria, such as *S. aureus*, remain in the CF airways even after HEMT.

We observed that other CF pathogens are not negatively impacted by LMW or HMW mucin to the degree observed for *S. aureus* (Fig. 6). Bacteria such as *P. aeruginosa* thrive in a mucus-rich environment and have developed ways to degrade mucin to benefit their growth and persistence (9, 58, 59). It was unsurprising to us that *P. aeruginosa* was able to grow in both LMW and HMW mucin (Fig. 6A and B) due to previous reports of *P. aeruginosa* proteases, including LasB and an unidentified metalloprotease, that can degrade mucin strands (25, 60). NTHi does not have any known mechanisms for the degradation of mucin polymer backbones, but it has a well-characterized sialidase that allows NTHi to utilize sialic acid residues from mucin side chains for biofilm maturation (61, 62). Interestingly, *S. epidermidis* was able to grow and persist in both LMW and HMW mucins, indicating that there are specific mechanisms that result in the inability of *S. aureus* to grow in the presence of LMW mucins.

Some limitations of this study include the inability to firmly control the size of the mucins in purchased BSM. Both BSM sources used contain a heterogeneous mixture of polymers of different lengths, with the HMW mucin having the widest range of polymer sizes. Other studies have noted that MUC5B monomer sizes average about 671 nm in length, and polymers measure approximately 11 µm with overall molecular weights ranging upward of 40 mDa (63–65). Additionally, BSM and PGM originating from animals may have batch-to-batch variation, and despite strict purification methods, there remains a possibility of contamination that must be addressed in studies using these products. Methods have been developed by other groups for collecting and purifying MUC5B from human saliva (66, 67). Utilizing native MUC5B mucin from human saliva in SCFM would give us a clearer understanding of how native mucins are impacting *S. aureus*. These interactions could be further characterized using a mucinase, such as StcE or bromelain, to cleave the mucin backbone for further testing of *S. aureus* survival (68–70). Other groups have found that degradation with trypsin and pepsin leads to degradation primarily at the non-glycosylated N-terminal site of mucin but do not fully reduce the size of mucin (71). Future studies would also benefit from examining differences in the mucin glycosylated side chains between the two available BSM sources used in this study and native MUC5B from human saliva to determine if a specific abundance of carbohydrate side chains negatively impacts *S. aureus* growth (8). Furthermore, our current model does not incorporate other antimicrobial factors that may be present at the epithelial surface, which will give important insights into how the epithelial layer may further impact *S. aureus* survival in the presence of mucins (72).

## MATERIALS AND METHODS

### Bacterial strains and growth conditions

*S. aureus* species (Table S1) were grown on tryptic soy broth (TSB) with 1.5% agar (TSA; BD Bioscience) with or without chloramphenicol (10 µg/mL; Millipore Sigma) to maintain plasmids in GFP-expressing strains at 37°C. Overnight cultures were inoculated from a single colony in 5 mL of TSB shaking at 200 RPM at 37°C. For GFP strains, chloramphenicol (10 µg/mL final concentration) was added to maintain plasmids when necessary. Synthetic cystic fibrosis sputum media (SCFM) was made as described in Palmer et al. SCFM variations included bovine submaxillary mucins, high molecular weight (Thermo Scientific; J63859.ME) or low molecular weight (Millipore Sigma; M3895-1G) at either 5 mg/mL or 2 mg/mL, and/or UltraPure Salmon Sperm DNA Solution (Invitrogen) at 0.2 mg/mL. Porcine gastric mucin (Millipore Sigma; M1778-10G) 5 mg/mL was dissolved in the SCFM base. For colony-forming units (CFUs), overnight cultures of bacteria were diluted 1:1 in fresh TSB and spun at 4,500 RPM to pellet bacteria. Bacteria were resuspended in 1× phosphate-buffered saline (PBS; Fischer Scientific). Approximately 1 × 10^7^ bacteria were inoculated per well of a 96-well flat-bottom plate (Greiner) containing 200 µL of SCFM with or without polymers. Plates were placed in an incubator, shaking at 37°C. CFUs were collected at 4, 8, 12, and 24 h and plated on TSA plates. Fluorescence-based growth curves were performed in a Spark Automated Multimode Microplate Reader (Tecan).

### LMW mucin filtration

Approximately 2 mL of sterile milliQ water was added to an Amicon Ultra centrifugal 100 kDa molecular weight cut-off filter (Millipore Sigma; UFC810008) and centrifuged (Eppendorf Centrifuge 5920 R) for 30 min at 3,428 relative centrifugal force (rcf) to wash before use; 2 mL of prepared LMW mucin in SCFM was added to the washed filter and centrifuged for 1 h at 3,428 rcf. The <100 kDa filtrate was collected and used for *S. aureus* survival assays.

### Transmission electron microscopy

SCFM with LMW or HMW mucin (5 mg/mL) was prepared and diluted to 100 µg/mL for TEM imaging. Samples were pipetted onto a carbon grid and allowed to incubate for one minute before washing with water and staining with 2% (wt/vol) uranyl acetate for one minute as previously reported (73). TEM images were acquired using a JEOL 1400 HC Flash microscope (JOEL USA).

### Fluorescence microscopy and quantification

Prior to inoculation with bacteria, 8-well glass-bottom chamber slides (Ibidi) were coated with 0.05% Poly-D-Lysine (Millipore Sigma) for 1 h, washed three times with filtered MilliQ water, and stored at 4°C until ready for use; 200 µL of SCFM was added per chamber, and approximately 1 × 10^7^ CFUs of GFP *S. aureus* was inoculated and allowed to grow statically for 24 h at 37°C prior to imaging. Slides were imaged on a Ti Eclipse widefield fluorescence microscope (Nikon). Biomass and aggregate size were quantified using the Nikon NIS-Elements AR software package (Version 5.42.02 Build 1801). Biomass quantification was performed on image Z-stacks after thresholding and 3D deconvolving, and aggregate sizes were determined by the NIS-elements object count function on maximum intensity projection images. Data analysis of biomass and aggregate size was performed in Microsoft Excel (Version 16.96.1 [25042021]). Aggregates smaller than 1 µm were excluded from data analysis to account for noise. Biomass measurements and aggregate sizes are representative of 3–5 independent experiments, with 3–6 individual fields of view measured for each sample for quantification.

### Statistical analyses

Statistical analyses were performed with GraphPad Prism version 10.4.2 (534) software (GraphPad by Dotmatrics). One-way analysis of variance (ANOVA) was performed on comparisons of CFUs at single time points, and two-way ANOVAs were performed on CFUs collected at multiple time points to determine significance between samples within a set time. Welch’s *t*-tests were performed on biomass and aggregate size. *P* values were considered significant if less than or equal to 0.05.

## SUPPLEMENTAL MATERIAL

Fig. S1 iai.00693-25-s0001.tiff LMW mucin and HMW mucin differ in terms of size.

Fig. S2 iai.00693-25-s0002.tiff Pig gastric Mucin does not inhibit *S. aureus* USA300.

Fig. S3 iai.00693-25-s0003.tiff LMW mucin filtrate containing molecules less than 100 kDa does not have anti-*S. aureus* activity.

Fig. S4 iai.00693-25-s0004.tiff Low molecular weight mucins reduce *S. aureus* USA100 survival over time.

Fig. S5 iai.00693-25-s0005.tiff Low molecular weight mucins reduce *S. aureus* USA100 biofilm biomass.

Fig. S6 iai.00693-25-s0006.tiff Reducing low molecular weight mucin concentration partially restores *S. aureus* USA100 growth and aggregate size.

Fig. S7 iai.00693-25-s0007.tiff Autofluorescence of SCFM with and without eDNA or mucin.

Fig. S8 iai.00693-25-s0008.tiff LMW mucin polymers broadly impact *S. aureus* laboratory and clinical isolate maximum endpoint GFP fluorescence intensity.

Fig. S9 iai.00693-25-s0009.tiff Growth of laboratory or clinical isolates of *S. aureus* in standard laboratory culture media with mucin.

Supplemental material iai.00693-25-s0010.docx Supplemental figure legends; Table S1.

ASM does not own the copyrights to Supplemental Material that may be linked to, or accessed through, an article. The authors have granted ASM a non-exclusive, world-wide license to publish the Supplemental Material files. Please contact the corresponding author directly for reuse.

## References

[1] Boucher RC. 2019. Muco-obstructive lung diseases. N Engl J Med 380:1941–1953. doi:10.1056/NEJMra1813799 31091375

[2] Morrison CB, Markovetz MR, Ehre C. 2019. Mucus, mucins, and cystic fibrosis. Pediatr Pulmonol 54:S84–S96. doi:10.1002/ppul.24530 31715083 PMC6853602

[3] Sheng YH, Hasnain SZ. 2022. Mucus and mucins: the underappreciated host defence system. Front Cell Infect Microbiol 12:856962. doi:10.3389/fcimb.2022.856962 35774401 PMC9238349

[4] Abdullah LH, Evans JR, Wang TT, Ford AA, Makhov AM, Nguyen K, Coakley RD, Griffith JD, Davis CW, Ballard ST, et al.. 2017. Defective postsecretory maturation of MUC5B mucin in cystic fibrosis airways. JCI Insight 2:e89752. doi:10.1172/jci.insight.89752 28352653 PMC5358479

[5] Okuda K, Chen G, Subramani DB, Wolf M, Gilmore RC, Kato T, Radicioni G, Kesimer M, Chua M, Dang H, et al.. 2019. Localization of secretory mucins MUC5AC and MUC5B in normal/healthy human airways. Am J Respir Crit Care Med 199:715–727. doi:10.1164/rccm.201804-0734OC 30352166 PMC6423099

[6] Ermund A, Meiss LN, Rodriguez-Pineiro AM, Bähr A, Nilsson HE, Trillo-Muyo S, Ridley C, Thornton DJ, Wine JJ, Hebert H, et al.. 2017. The normal trachea is cleaned by MUC5B mucin bundles from the submucosal glands coated with the MUC5AC mucin. Biochem Biophys Res Commun 492:331–337. doi:10.1016/j.bbrc.2017.08.113 28859985 PMC5596833

[7] Ridley C, Kouvatsos N, Raynal BD, Howard M, Collins RF, Desseyn J-L, Jowitt TA, Baldock C, Davis CW, Hardingham TE, et al.. 2014. Assembly of the respiratory mucin MUC5B. A new model for a gel-forming mucin. J Biol Chem 289:16409–16420. doi:10.1074/jbc.M114.566679 24778189 PMC4047408

[8] Bej R, Haag R. 2022. Mucus-inspired dynamic hydrogels: synthesis and future perspectives. J Am Chem Soc 144:20137–20152. doi:10.1021/jacs.1c13547 36074739 PMC9650700

[9] Arif SJ, Hoffman KM, Flynn JM, Wiggen TD, Lucas SK, Villarreal AR, Gilbertsen AJ, Dunitz JM, Hunter RC. 2025. Host- and microbial-mediated mucin degradation differentially shape Pseudomonas aeruginosa physiology and gene expression. PLoS Pathog 21:e1013568. doi:10.1371/journal.ppat.1013568 41042816 PMC12503327

[10] Henke MO, John G, Rheineck C, Chillappagari S, Naehrlich L, Rubin BK. 2011. Serine proteases degrade airway mucins in cystic fibrosis. Infect Immun 79:3438–3444. doi:10.1128/IAI.01252-10 21646446 PMC3147599

[11] Shull LM, Wolter DJ, Kunkle DE, Legg KA, Giedroc DP, Skaar EP, Hoffman LR, Reniere ML. 2025. Analysis of genetic requirements and nutrient availability for Staphylococcus aureus growth in cystic fibrosis sputum. bioRxiv. doi:10.1101/2024.09.24.614743 PMC12077221 40172197

[12] Palmer KL, Aye LM, Whiteley M. 2007. Nutritional cues control Pseudomonas aeruginosa multicellular behavior in cystic fibrosis sputum. J Bacteriol 189:8079–8087. doi:10.1128/JB.01138-07 17873029 PMC2168676

[13] Matsui H, Wagner VE, Hill DB, Schwab UE, Rogers TD, Button B, Taylor RM II, Superfine R, Rubinstein M, Iglewski BH, et al.. 2006. A physical linkage between cystic fibrosis airway surface dehydration and Pseudomonas aeruginosa biofilms. Proc Natl Acad Sci USA 103:18131–18136. doi:10.1073/pnas.0606428103 17116883 PMC1838718

[14] Deinhardt-Emmer S, Sachse S, Geraci J, Fischer C, Kwetkat A, Dawczynski K, Tuchscherr L, Löffler B. 2018. Virulence patterns of Staphylococcus aureus strains from nasopharyngeal colonization. J Hosp Infect 100:309–315. doi:10.1016/j.jhin.2017.12.011 29253623

[15] Wertheim HFL, Melles DC, Vos MC, van Leeuwen W, van Belkum A, Verbrugh HA, Nouwen JL. 2005. The role of nasal carriage in Staphylococcus aureus infections. Lancet Infect Dis 5:751–762. doi:10.1016/S1473-3099(05)70295-4 16310147

[16] Roden L, Görlich D, Omran H, Peters G, Große-Onnebrink J, Kahl BC. 2019. A retrospective analysis of the pathogens in the airways of patients with primary ciliary dyskinesia. Respir Med 156:69–77. doi:10.1016/j.rmed.2019.08.009 31437650

[17] Cystic Fibrosis Foundation. 2019. Patient registry annual data report.

[18] Luo L, Tang J, Du X, Li N. 2024. Chronic obstructive pulmonary disease and the airway microbiome: a review for clinicians. Respir Med 225:107586. doi:10.1016/j.rmed.2024.107586 38460708

[19] Lucas SK, Villarreal AR, Ahmad MM, Itabiyi A, Feddema E, Boyer HC, Hunter RC. 2021. Anaerobic microbiota derived from the upper airways impact Staphylococcus aureus physiology. Infect Immun 89:e0015321. doi:10.1128/IAI.00153-21 34125598 PMC8370671

[20] Shuter J, Hatcher VB, Lowy FD. 1996. Staphylococcus aureus binding to human nasal mucin. Infect Immun 64:310–318. doi:10.1128/iai.64.1.310-318.1996 8557357 PMC173761

[21] Nichols DP, Paynter AC, Heltshe SL, Donaldson SH, Frederick CA, Freedman SD, Gelfond D, Hoffman LR, Kelly A, Narkewicz MR, et al.. 2022. Clinical effectiveness of elexacaftor/tezacaftor/ivacaftor in people with cystic fibrosis: a clinical trial. Am J Respir Crit Care Med 205:529–539. doi:10.1164/rccm.202108-1986OC 34784492 PMC8906485

[22] Morgan SJ, Coulter E, Betts HL, Solomon GM, Clancy JP, Rowe SM, Nichols DP, Singh PK. 2024. Elexacaftor/tezacaftor/ivacaftor’s effects on cystic fibrosis infections are maintained, but not increased, after 3.5 years of treatment. J Clin Invest 134:e184171. doi:10.1172/JCI184171 39235967 PMC11473140

[23] Reihill JA, Douglas LEJ, Martin SL. 2021. Modulation of ion transport to restore airway hydration in cystic fibrosis. Genes (Basel) 12:453. doi:10.3390/genes12030453 33810137 PMC8004921

[24] Turner KH, Wessel AK, Palmer GC, Murray JL, Whiteley M. 2015. Essential genome of Pseudomonas aeruginosa in cystic fibrosis sputum. Proc Natl Acad Sci USA 112:4110–4115. doi:10.1073/pnas.1419677112 25775563 PMC4386324

[25] Costello C, Murphree-Terry M, Oden A, Combs S, Keith J, Birket S. 2025. Pseudomonas aeruginosa increases viscoelasticity and decreases transportability of artificial mucus. iScience 28:113265. doi:10.1016/j.isci.2025.113265 40837233 PMC12362397

[26] Lewin GR, Kapur A, Cornforth DM, Duncan RP, Diggle FL, Moustafa DA, Harrison SA, Skaar EP, Chazin WJ, Goldberg JB, et al.. 2023. Application of a quantitative framework to improve the accuracy of a bacterial infection model. Proc Natl Acad Sci USA 120:e2221542120. doi:10.1073/pnas.2221542120 37126703 PMC10175807

[27] Cornforth DM, Diggle FL, Melvin JA, Bomberger JM, Whiteley M. 2020. Quantitative framework for model evaluation in microbiology research using Pseudomonas aeruginosa and cystic fibrosis infection as a test case. mBio 11:e03042-19. doi:10.1128/mBio.03042-19 31937646 PMC6960289

[28] Tirelli C, Mira S, Belmonte LA, De Filippi F, De Grassi M, Italia M, Maggioni S, Guido G, Mondoni M, Canonica GW, et al.. 2024. Exploring the potential role of metabolomics in COPD: a concise review. Cells 13:475. doi:10.3390/cells13060475 38534319 PMC10969696

[29] Montuschi P, Paris D, Montella S, Melck D, Mirra V, Santini G, Mores N, Montemitro E, Majo F, Lucidi V, et al.. 2014. Nuclear magnetic resonance–based metabolomics discriminates primary ciliary dyskinesia from cystic fibrosis. Am J Respir Crit Care Med 190:229–233. doi:10.1164/rccm.201402-0249LE 25025356

[30] Kim J, Lee J, Jang Y, Ha J, Kim D, Ji M, Lee YK, Kim W, You S, Do J, et al.. 2019. N-glycans of bovine submaxillary mucin contain core-fucosylated and sulfated glycans but not sialylated glycans. Int J Biol Macromol 138:1072–1078. doi:10.1016/j.ijbiomac.2019.07.108 31325506

[31] Madsen JB, Pakkanen KI, Duelund L, Svensson B, Hachem MA, Lee S. 2015. A simplified chromatographic approach to purify commercially available bovine submaxillary mucins (BSM). Prep Biochem Biotechnol 45:84–99. doi:10.1080/10826068.2014.887583 24547990

[32] Henry RL, Mellis CM, Petrovic L. 1992. Mucoid Pseudomonas aeruginosa is a marker of poor survival in cystic fibrosis. Pediatr Pulmonol 12:158–161. doi:10.1002/ppul.1950120306 1641272

[33] Pillarisetti N, Williamson E, Linnane B, Skoric B, Robertson CF, Robinson P, Massie J, Hall GL, Sly P, Stick S, et al.. 2011. Infection, inflammation, and lung function decline in infants with cystic fibrosis. Am J Respir Crit Care Med 184:75–81. doi:10.1164/rccm.201011-1892OC 21493738

[34] Filkins LM, Hampton TH, Gifford AH, Gross MJ, Hogan DA, Sogin ML, Morrison HG, Paster BJ, O’Toole GA. 2012. Prevalence of streptococci and increased polymicrobial diversity associated with cystic fibrosis patient stability. J Bacteriol 194:4709–4717. doi:10.1128/JB.00566-12 22753064 PMC3415522

[35] Cuthbertson L, Walker AW, Oliver AE, Rogers GB, Rivett DW, Hampton TH, Ashare A, Elborn JS, De Soyza A, Carroll MP, et al.. 2020. Lung function and microbiota diversity in cystic fibrosis. Microbiome 8:45. doi:10.1186/s40168-020-00810-3 32238195 PMC7114784

[36] Donaldson SH, Laube BL, Corcoran TE, Bhambhvani P, Zeman K, Ceppe A, Zeitlin PL, Mogayzel PJ, Boyle M, Locke LW, et al.. 2018. Effect of ivacaftor on mucociliary clearance and clinical outcomes in cystic fibrosis patients with G551D-CFTR. JCI Insight 3:e122695. doi:10.1172/jci.insight.122695 30568035 PMC6338313

[37] Dittrich A-M, Sieber S, Naehrlich L, Burkhart M, Hafkemeyer S, Tümmler B. 2024. Use of elexacaftor/tezacaftor/ivacaftor leads to changes in detection frequencies of Staphylococcus aureus and Pseudomonas aeruginosa dependent on age and lung function in people with cystic fibrosis. Int J Infect Dis 139:124–131. doi:10.1016/j.ijid.2023.11.013 38036261

[38] Kato T, Radicioni G, Papanikolas MJ, Stoychev GV, Markovetz MR, Aoki K, Porterfield M, Okuda K, Barbosa Cardenas SM, Gilmore RC, et al.. 2022. Mucus concentration–dependent biophysical abnormalities unify submucosal gland and superficial airway dysfunction in cystic fibrosis. Sci Adv 8:eabm9718. doi:10.1126/sciadv.abm9718 35363522 PMC10938572

[39] Esther CR, Muhlebach MS, Ehre C, Hill DB, Wolfgang MC, Kesimer M, Ramsey KA, Markovetz MR, Garbarine IC, Forest MG, et al.. 2019. Mucus accumulation in the lungs precedes structural changes and infection in children with cystic fibrosis. Sci Transl Med 11:eaav3488. doi:10.1126/scitranslmed.aav3488 30944166 PMC6566903

[40] Kim J, Ryu C, Ha J, Lee J, Kim D, Ji M, Park CS, Lee J, Kim DK, Kim HH. 2020. Structural and quantitative characterization of mucin-type O-glycans and the identification of O-glycosylation sites in bovine submaxillary mucin. Biomolecules 10:636. doi:10.3390/biom10040636 32326134 PMC7226346

[41] Zarepour M, Bhullar K, Montero M, Ma C, Huang T, Velcich A, Xia L, Vallance BA. 2013. The mucin Muc2 limits pathogen burdens and epithelial barrier dysfunction during Salmonella enterica serovar Typhimurium colitis. Infect Immun 81:3672–3683. doi:10.1128/IAI.00854-13 23876803 PMC3811786

[42] Batson BD, Zorn BT, Radicioni G, Livengood SS, Kumagai T, Dang H, Ceppe A, Clapp PW, Tunney M, et al.. 2022. Cystic fibrosis airway mucus hyperconcentration produces a vicious cycle of mucin, pathogen, and inflammatory interactions that promotes disease persistence. Am J Respir Cell Mol Biol 67:253–265. doi:10.1165/rcmb.2021-0359OC 35486871 PMC9348562

[43] Ibberson CB, Whiteley M. 2019. The Staphylococcus aureus transcriptome during cystic fibrosis lung infection. mBio 10:e02774-19. doi:10.1128/mBio.02774-19 31744924 PMC6867902

[44] Boles BR, Horswill AR. 2008. agr-mediated dispersal of Staphylococcus aureus biofilms. PLoS Pathog 4:e1000052. doi:10.1371/journal.ppat.1000052 18437240 PMC2329812

[45] Mann EE, Rice KC, Boles BR, Endres JL, Ranjit D, Chandramohan L, Tsang LH, Smeltzer MS, Horswill AR, Bayles KW. 2009. Modulation of eDNA release and degradation affects Staphylococcus aureus biofilm maturation. PLoS One 4:e5822. doi:10.1371/journal.pone.0005822 19513119 PMC2688759

[46] Rice KC, Mann EE, Endres JL, Weiss EC, Cassat JE, Smeltzer MS, Bayles KW. 2007. The cidA murein hydrolase regulator contributes to DNA release and biofilm development in Staphylococcus aureus. Proc Natl Acad Sci USA 104:8113–8118. doi:10.1073/pnas.0610226104 17452642 PMC1876580

[47] Kiedrowski MR, Kavanaugh JS, Malone CL, Mootz JM, Voyich JM, Smeltzer MS, Bayles KW, Horswill AR. 2011. Nuclease modulates biofilm formation in community-associated methicillin-resistant Staphylococcus aureus. PLoS One 6:e26714. doi:10.1371/journal.pone.0026714 22096493 PMC3214024

[48] Ding X, Robbe-Masselot C, Fu X, Léonard R, Marsac B, Dauriat CJG, Lepissier A, Rytter H, Ramond E, Dupuis M, et al.. 2023. Airway environment drives the selection of quorum sensing mutants and promote Staphylococcus aureus chronic lifestyle. Nat Commun 14:8135. doi:10.1038/s41467-023-43863-2 38065959 PMC10709412

[49] Smyth DS, Kafer JM, Wasserman GA, Velickovic L, Mathema B, Holzman RS, Knipe TA, Becker K, von Eiff C, Peters G, et al.. 2012. Nasal carriage as a source of agr-defective Staphylococcus aureus bacteremia. J Infect Dis 206:1168–1177. doi:10.1093/infdis/jis483 22859823 PMC3448967

[50] Parker CP, Akil N, Shanrock CR, Allen PD, Chaly AL, Crosby HA, Kwiecinski J, Horswill AR, Fischer AJ. 2020. MgrA regulates interaction of Staphylococcus aureus with mucin. bioRxiv. doi:10.1101/2020.04.09.035261

[51] Crosby HA, Schlievert PM, Merriman JA, King JM, Salgado-Pabón W, Horswill AR. 2016. The Staphylococcus aureus global regulator MgrA modulates clumping and virulence by controlling surface protein expression. PLoS Pathog 12:e1005604. doi:10.1371/journal.ppat.1005604 27144398 PMC4856396

[52] Li L, Wang G, Cheung A, Abdelhady W, Seidl K, Xiong YQ. 2019. MgrA governs adherence, host cell interaction, and virulence in a murine model of bacteremia due to Staphylococcus aureus. J Infect Dis 220:1019–1028. doi:10.1093/infdis/jiz219 31177268 PMC6688059

[53] Walker JN, Crosby HA, Spaulding AR, Salgado-Pabón W, Malone CL, Rosenthal CB, Schlievert PM, Boyd JM, Horswill AR. 2013. The Staphylococcus aureus ArlRS two-component system is a novel regulator of agglutination and pathogenesis. PLoS Pathog 9:e1003819. doi:10.1371/journal.ppat.1003819 24367264 PMC3868527

[54] Queck SY, Jameson-Lee M, Villaruz AE, Bach T-HL, Khan BA, Sturdevant DE, Ricklefs SM, Li M, Otto M. 2008. RNAIII-independent target gene control by the agr quorum-sensing system: insight into the evolution of virulence regulation in Staphylococcus aureus. Mol Cell 32:150–158. doi:10.1016/j.molcel.2008.08.005 18851841 PMC2575650

[55] Gupta RK, Luong TT, Lee CY. 2015. RNAIII of the Staphylococcus aureus agr system activates global regulator MgrA by stabilizing mRNA. Proc Natl Acad Sci USA 112:14036–14041. doi:10.1073/pnas.1509251112 26504242 PMC4653210

[56] Jacob KM, Hernández-Villamizar S, Hammer ND, Reguera G. 2024. Mucin-induced surface dispersal of Staphylococcus aureus and Staphylococcus epidermidis via quorum-sensing dependent and independent mechanisms. mBio 15:e0156224. doi:10.1128/mbio.01562-24 38953351 PMC11323471

[57] Huffines JT, Kiedrowski MR. 2025. Staphylococcus aureus phenol-soluble modulins have dispersal and anti-aggregation activity towards corynebacteria. J Bacteriol 207:e0018325. doi:10.1128/jb.00183-25 40810517 PMC12445096

[58] Aristoteli LP, Willcox MDP. 2003. Mucin degradation mechanisms by distinct Pseudomonas aeruginosa isolates in vitro. Infect Immun 71:5565–5575. doi:10.1128/IAI.71.10.5565-5575.2003 14500475 PMC201046

[59] Hoffman CL, Lalsiamthara J, Aballay A. 2020. Host mucin is exploited by Pseudomonas aeruginosa to provide monosaccharides required for a successful infection. mBio 11:e00060-20. doi:10.1128/mBio.00060-20 32127446 PMC7064748

[60] Bever RA, Iglewski BH. 1988. Molecular characterization and nucleotide sequence of the Pseudomonas aeruginosa elastase structural gene. J Bacteriol 170:4309–4314. doi:10.1128/jb.170.9.4309-4314.1988 2842313 PMC211443

[61] Langereis JD, Hermans PWM. 2013. Novel concepts in nontypeable Haemophilus influenzae biofilm formation. FEMS Microbiol Lett 346:81–89. doi:10.1111/1574-6968.12203 23808954

[62] Ng PSK, Day CJ, Atack JM, Hartley-Tassell LE, Winter LE, Marshanski T, Padler-Karavani V, Varki A, Barenkamp SJ, Apicella MA, et al.. 2019. Nontypeable Haemophilus influenzae has evolved preferential use of N-acetylneuraminic acid as a host adaptation. mBio 10:e00422-19. doi:10.1128/mBio.00422-19 31064827 PMC6509186

[63] Hughes GW, Ridley C, Collins R, Roseman A, Ford R, Thornton DJ. 2019. The MUC5B mucin polymer is dominated by repeating structural motifs and its topology is regulated by calcium and pH. Sci Rep 9:17350. doi:10.1038/s41598-019-53768-0 31758042 PMC6874590

[64] Oppenheim FG, Salih E, Siqueira WL, Zhang W, Helmerhorst EJ. 2007. Salivary proteome and its genetic polymorphisms. Ann N Y Acad Sci 1098:22–50. doi:10.1196/annals.1384.030 17303824

[65] Loomis RE, Prakobphol A, Levine MJ, Reddy MS, Jones PC. 1987. Biochemical and biophysical comparison of two mucins from human submandibular-sublingual saliva. Arch Biochem Biophys 258:452–464. doi:10.1016/0003-9861(87)90366-3 3674885

[66] Veerman EC, van den Keybus PA, Valentijn-Benz M, Nieuw Amerongen AV. 1992. Isolation of different high-M_r_ mucin species from human whole saliva. Biochem J 283:807–811. doi:10.1042/bj2830807 1590770 PMC1130958

[67] Rousseau K, Kirkham S, Johnson L, Fitzpatrick B, Howard M, Adams EJ, Rogers DF, Knight D, Clegg P, Thornton DJ. 2008. Proteomic analysis of polymeric salivary mucins: no evidence for MUC19 in human saliva. Biochem J 413:545–552. doi:10.1042/BJ20080260 18426393

[68] Malaker SA, Pedram K, Ferracane MJ, Bensing BA, Krishnan V, Pett C, Yu J, Woods EC, Kramer JR, Westerlind U, et al.. 2019. The mucin-selective protease StcE enables molecular and functional analysis of human cancer-associated mucins. Proc Natl Acad Sci USA 116:7278–7287. doi:10.1073/pnas.1813020116 30910957 PMC6462054

[69] Pereira de Sousa I, Cattoz B, Wilcox MD, Griffiths PC, Dalgliesh R, Rogers S, Bernkop-Schnürch A. 2015. Nanoparticles decorated with proteolytic enzymes, a promising strategy to overcome the mucus barrier. Eur J Pharm Biopharm 97:257–264. doi:10.1016/j.ejpb.2015.01.008 25661320

[70] Valle N, Eapen MS, Pillai K, Morris R, Akhter J, Mekkawy AH, Morris DL, Valle SJ. 2024. Impact of nebulized BromAc on mucus plug clearance in a mechanically ventilated ex vivo ovine lung model of obstructive respiratory conditions. Life (Basel) 14:1111. doi:10.3390/life14091111 39337895 PMC11433166

[71] Madsen JB, Svensson B, Abou Hachem M, Lee S. 2015. Proteolytic degradation of bovine submaxillary mucin (BSM) and its impact on adsorption and lubrication at a hydrophobic surface. Langmuir 31:8303–8309. doi:10.1021/acs.langmuir.5b01281 26153254

[72] Ramalho AS, Amato F, Gentzsch M. 2023. Patient-derived cell models for personalized medicine approaches in cystic fibrosis. J Cyst Fibros 22:S32–S38. doi:10.1016/j.jcf.2022.11.007 36529661 PMC9992303

[73] Harris ES, McIntire HJ, Mazur M, Schulz-Hildebrandt H, Leung HM, Tearney GJ, Krick S, Rowe SM, Barnes JW. 2024. Reduced sialylation of airway mucin impairs mucus transport by altering the biophysical properties of mucin. Sci Rep 14:16568. doi:10.1038/s41598-024-66510-2 39019950 PMC11255327

