# Supplementary figures and images for: Increased low-molecular-weight mucins in muco-obstructive airway disease limit Staphylococcus aureus growth
Source: Infect Immun. 2026 Jun 4;94(7):e00693-25. doi: 10.1128/iai.00693-25 (PMC13367046; doi:10.1128/iai.00693-25)

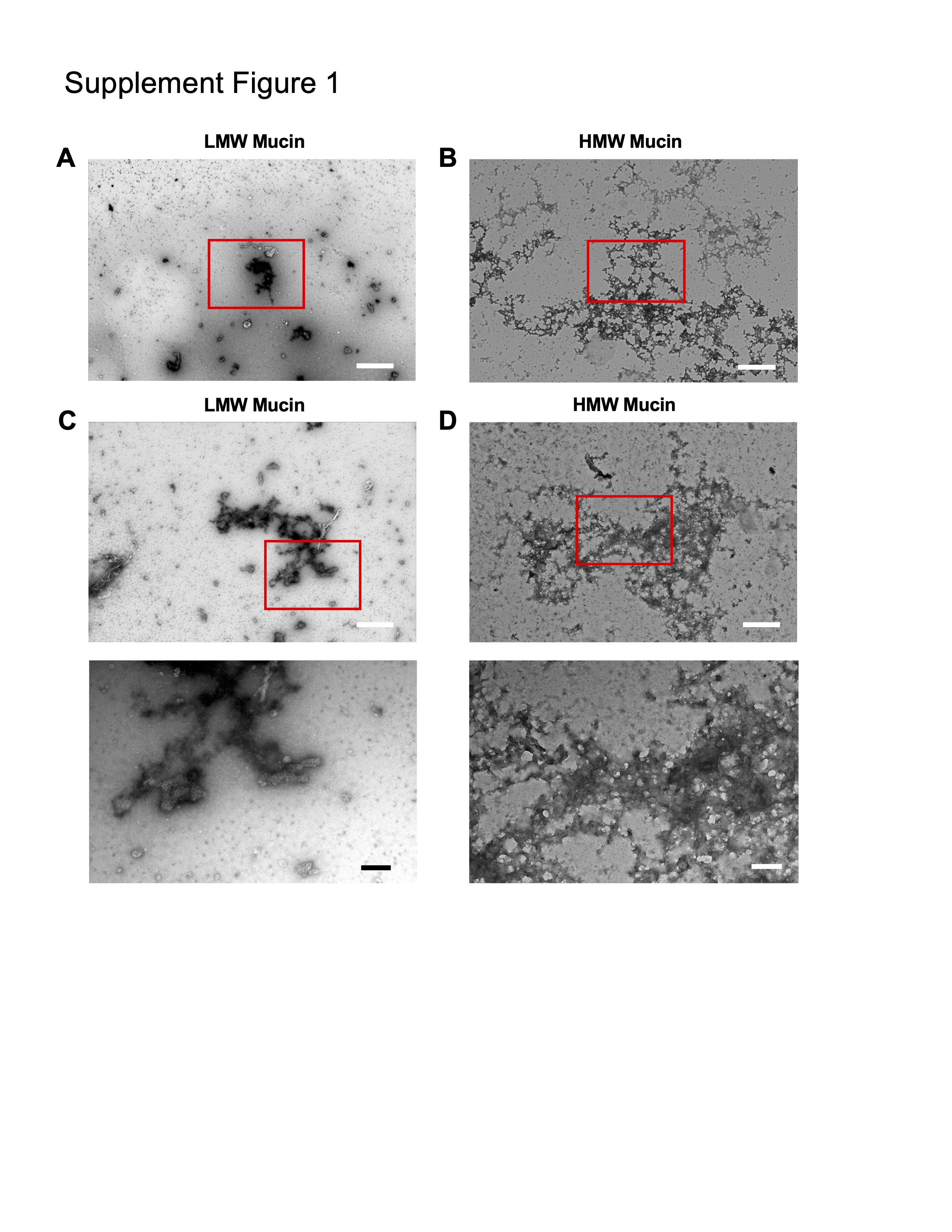

Supplement: Fig. S1 — LMW mucin and HMW mucin differ in terms of size. [file iai.00693-25-s0001.tiff]

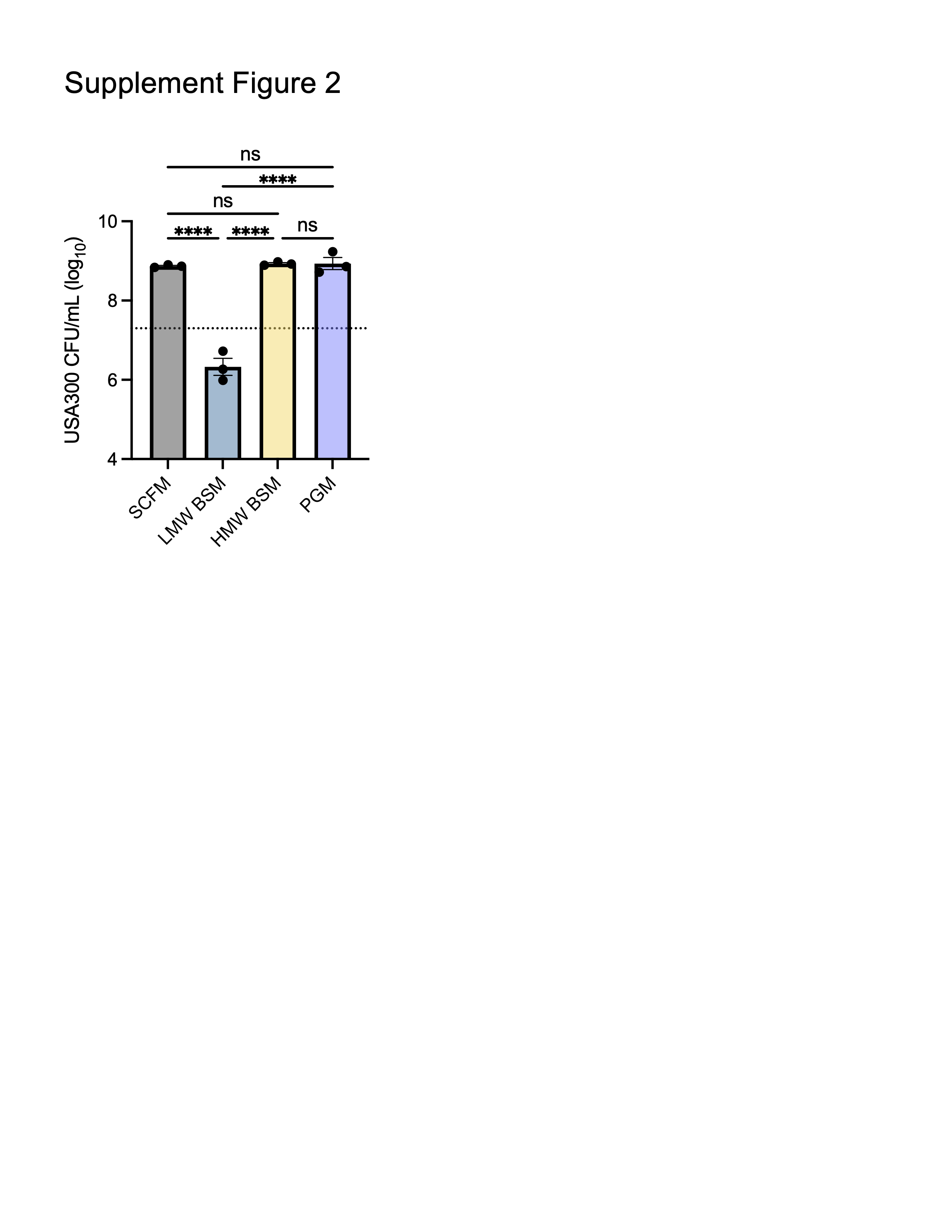

Supplement: Fig. S2 — Pig gastric Mucin does not inhibit S. aureus USA300. [file iai.00693-25-s0002.tiff]

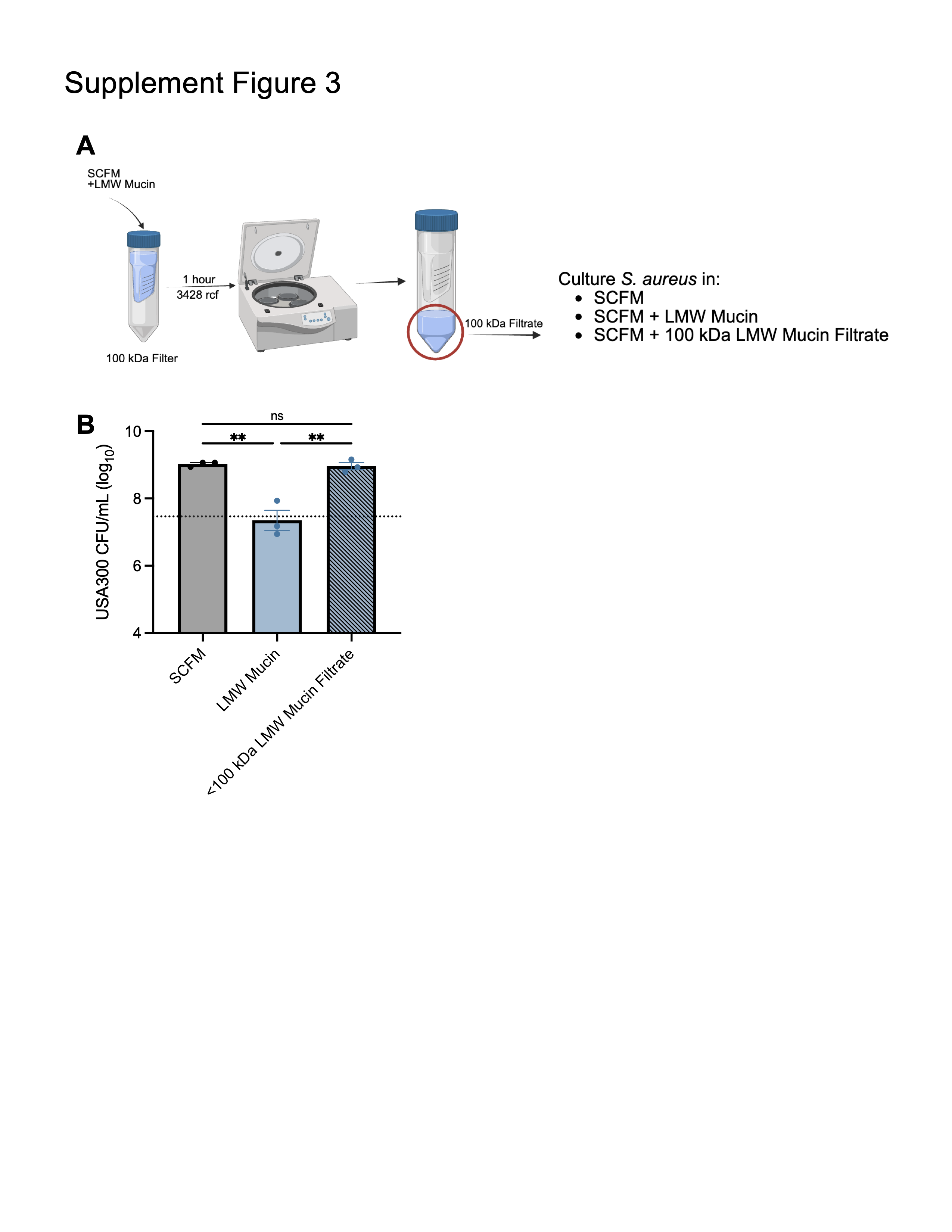

Supplement: Fig. S3 — LMW mucin filtrate containing molecules less than 100 kDa does not have anti-S. aureus activity. [file iai.00693-25-s0003.tiff]

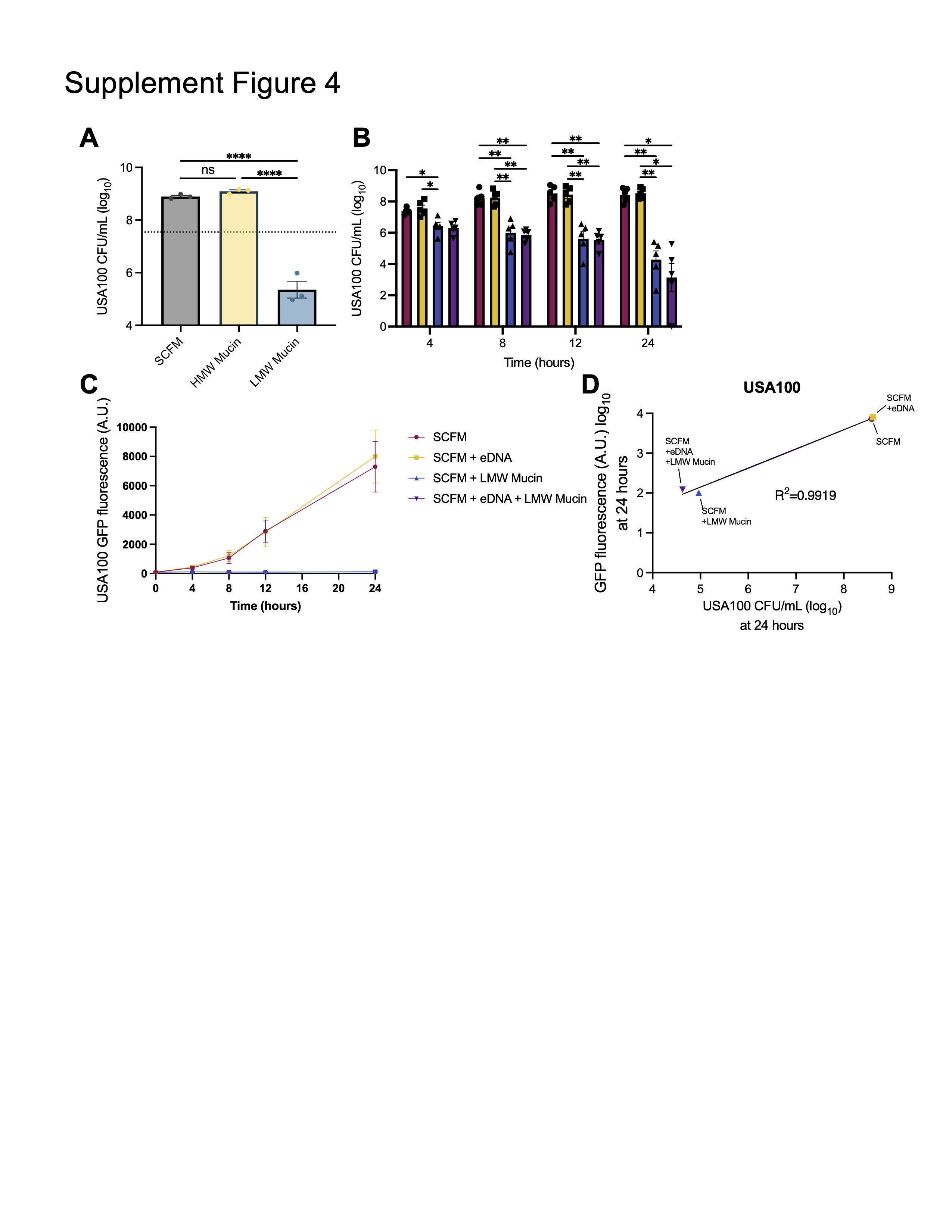

Supplement: Fig. S4 — Low molecular weight mucins reduce S. aureus USA100 survival over time. [file iai.00693-25-s0004.tiff]

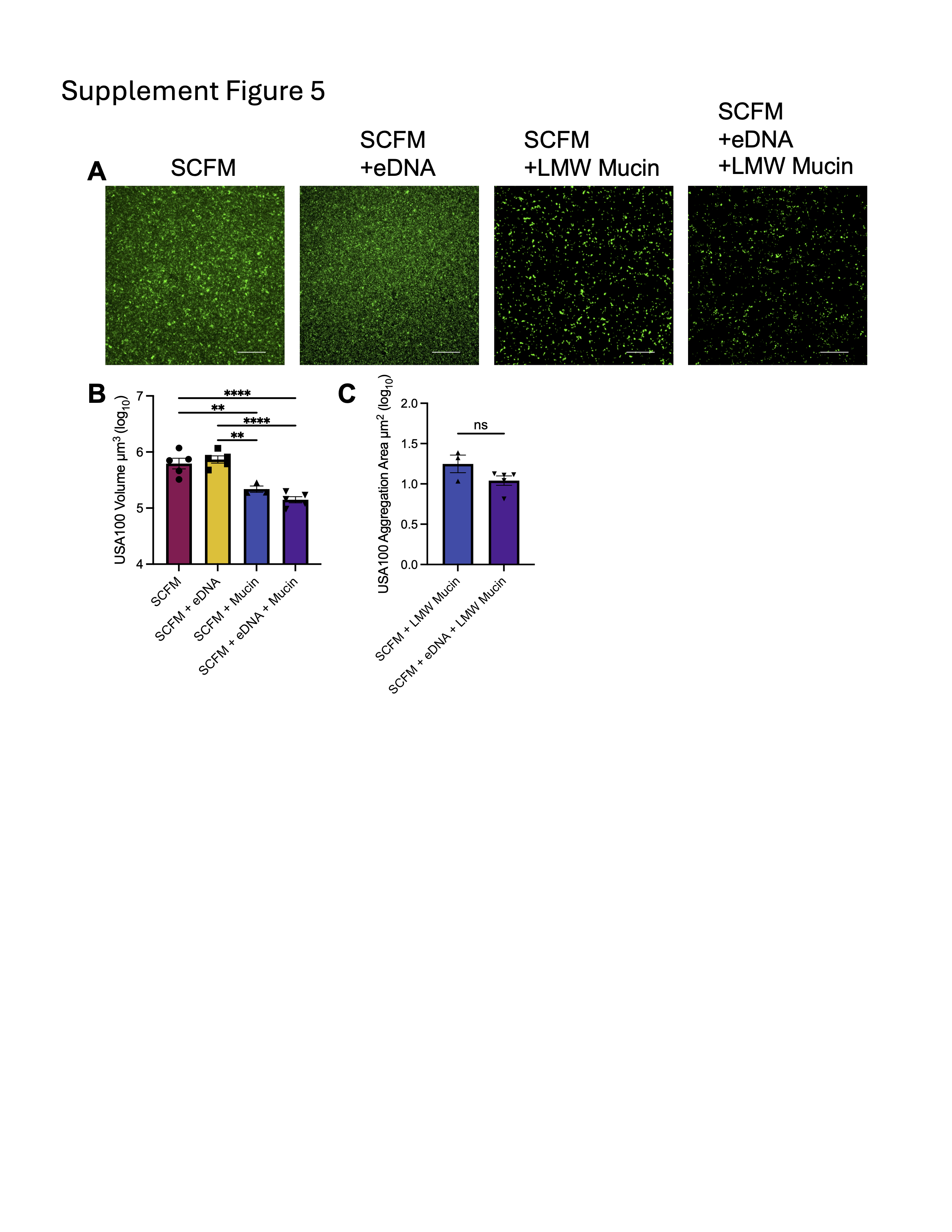

Supplement: Fig. S5 — Low molecular weight mucins reduce S. aureus USA100 biofilm biomass. [file iai.00693-25-s0005.tiff]

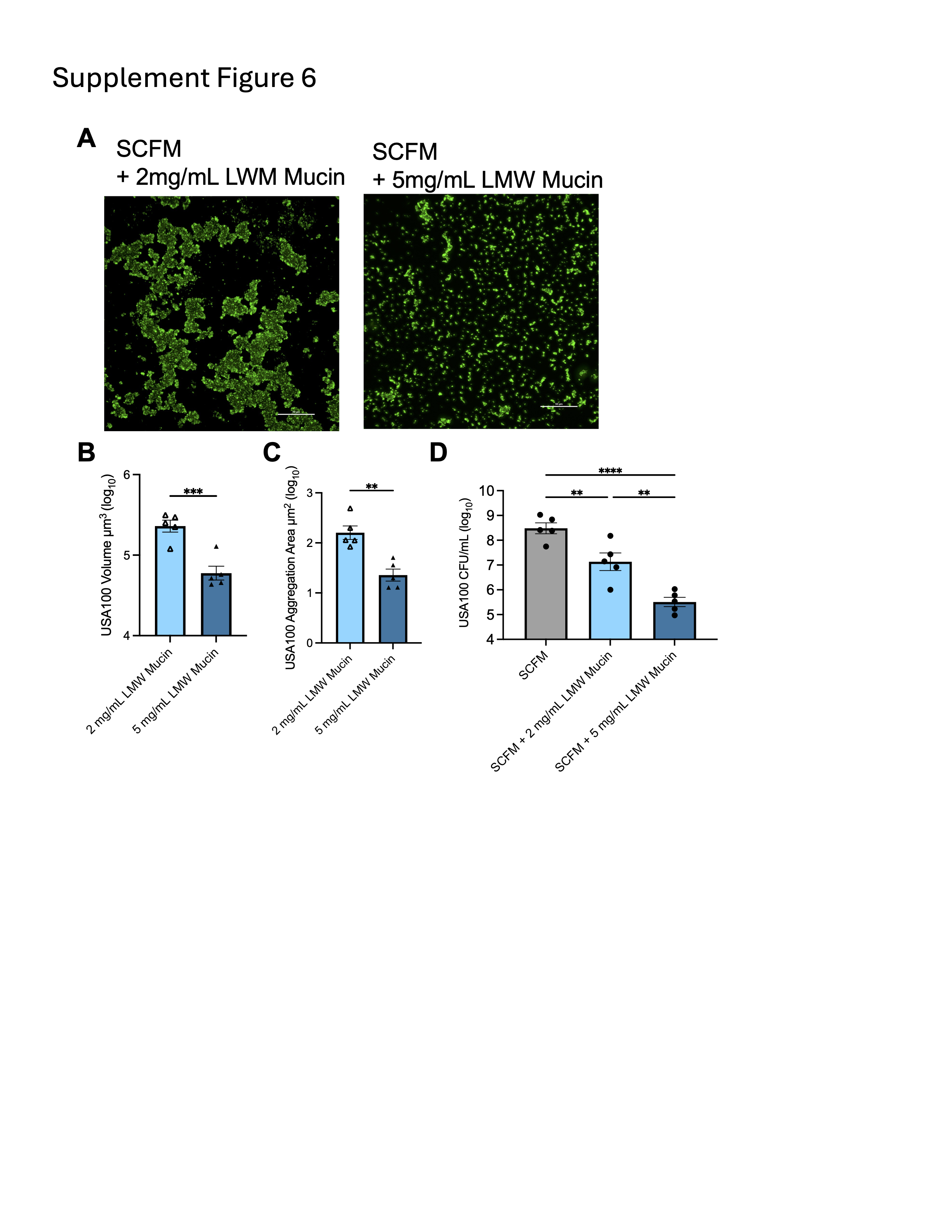

Supplement: Fig. S6 — Reducing low molecular weight mucin concentration partially restores S. aureus USA100 growth and aggregate size. [file iai.00693-25-s0006.tiff]

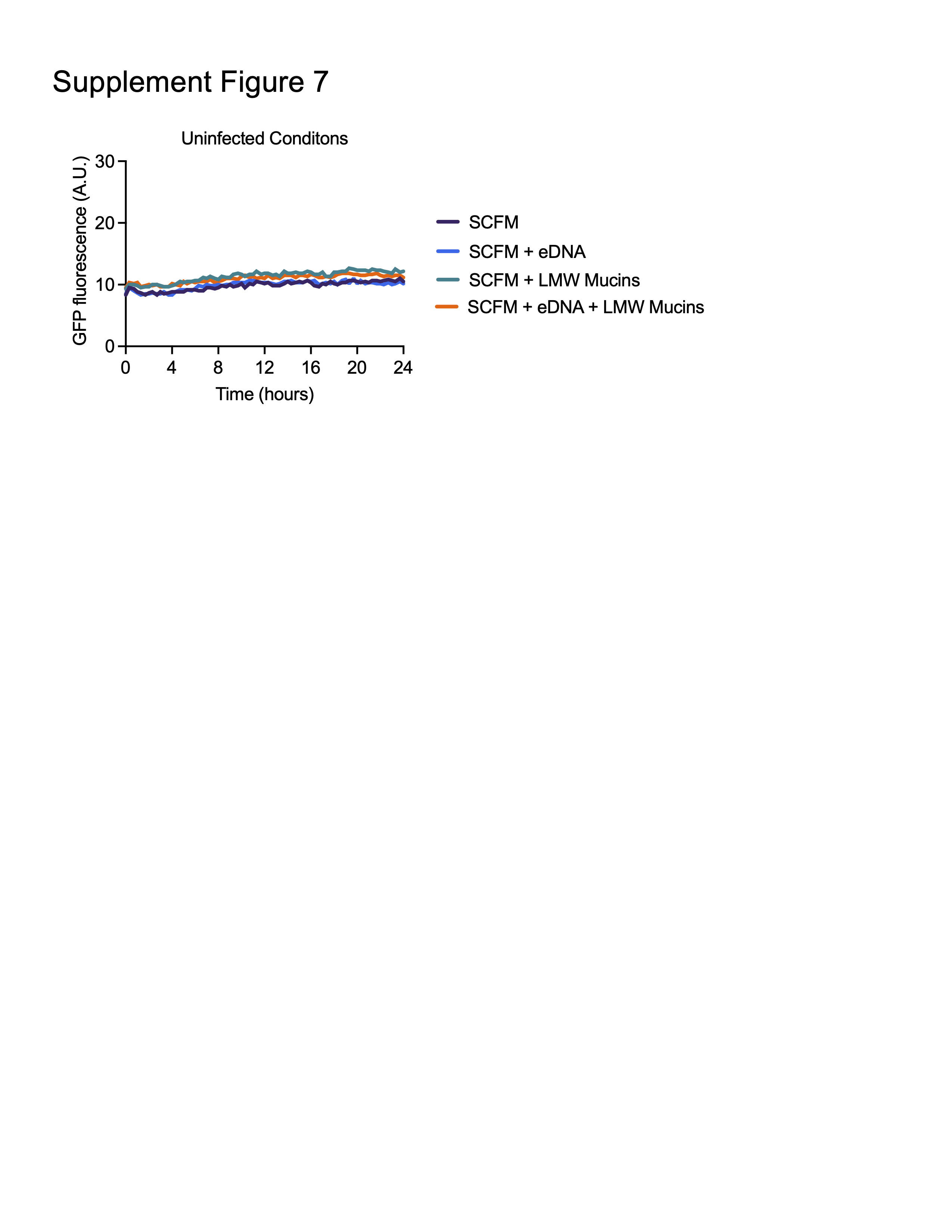

Supplement: Fig. S7 — Autofluorescence of SCFM with and without eDNA or mucin. [file iai.00693-25-s0007.tiff]

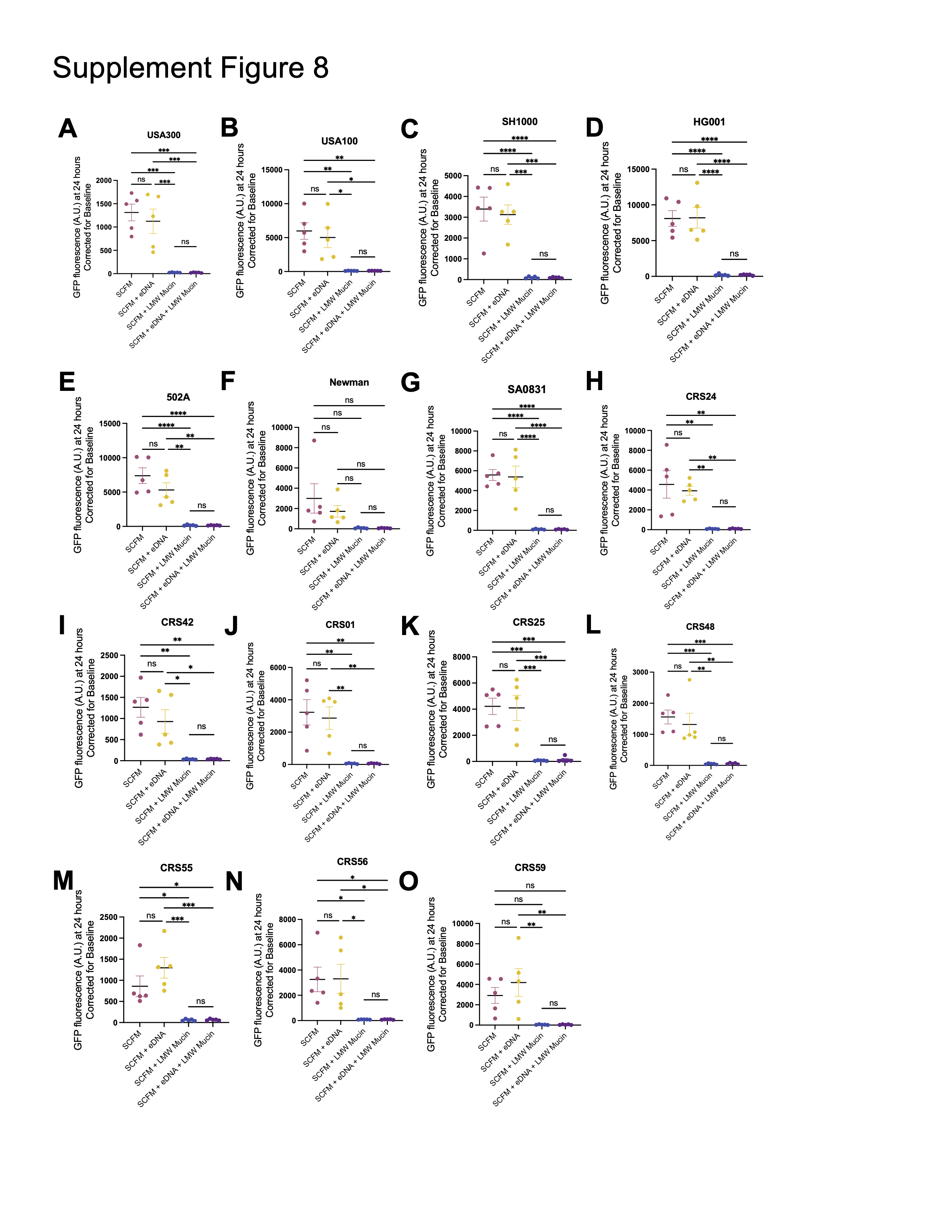

Supplement: Fig. S8 — LMW mucin polymers broadly impact S. aureus laboratory and clinical isolate maximum endpoint GFP fluorescence intensity. [file iai.00693-25-s0008.tiff]

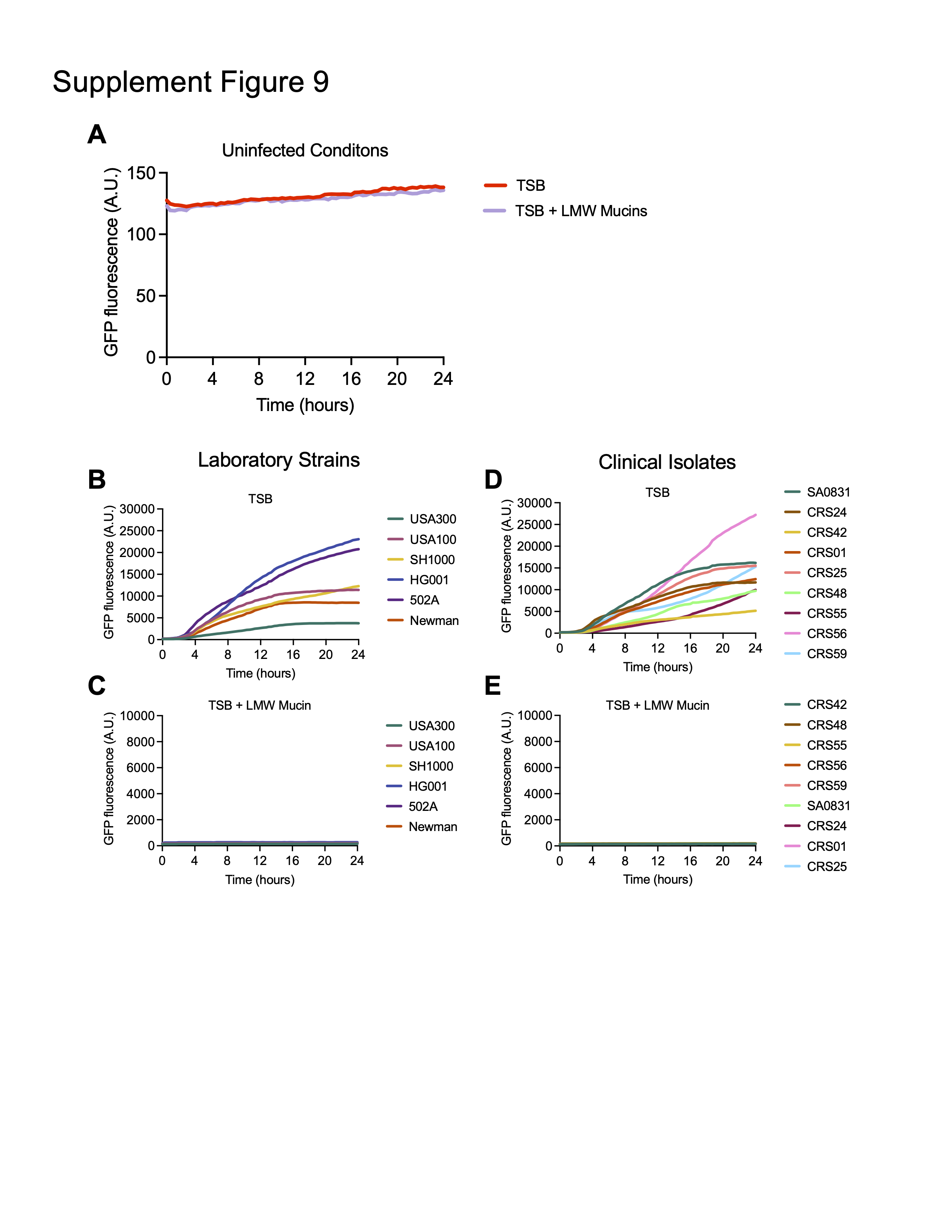

Supplement: Fig. S9 — Growth of laboratory or clinical isolates of S. aureus in standard laboratory culture media with mucin. [file iai.00693-25-s0009.tiff]
