# Supplementary material for: Increased low-molecular-weight mucins in muco-obstructive airway disease limit Staphylococcus aureus growth
Source: Infect Immun. 2026 Jun 4;94(7):e00693-25. doi: 10.1128/iai.00693-25 (PMC13367046; doi:10.1128/iai.00693-25)
Supplement: Supplemental material — Supplemental figure legends; Table S1. [file iai.00693-25-s0010.docx]

**SUPPORTING INFORMATION**

**SUPPLEMENTAL FIGURE LEGENDS**

**Figure S1: LMW mucin and HMW mucin differ in terms of size.** Transmission electron microscopy of LMW mucin (left) and HMW mucin (right). Zoomed out images (A and B) of Figure 1B and C (indicated by red box). Scale bar represents 800 nm. Additional fields (C and D) of LMW mucin (Left) and HMW mucin (right) at 800 nm (top) and 200 nm (bottom).

**Figure S2: Pig gastric Mucin does not inhibit *S. aureus* USA300.** USA300 CFUs in SCFM (grey), SCFM + LMW BSM (blue), SCFM + HMW Mucin (yellow), or SCFM + PGM (purple) at 24 hours. One-way ANOVA was performed on USA300 CFUs. (****P < 0.0001, ns = nonsignificant). Data is represented by mean ± SEM. Dotted line represents average inoculum of USA300.

**Figure S3: LMW mucin filtrate containing molecules less than 100kDa does not have anti-*S. aureus* activity.** Experimental schematic of mucin filtration. (A) USA300 CFUs (B) in LWM supernatant media in <100 kDa after 24 hours of growth. One-way ANOVA was performed on USA300 colony-forming units (CFUs) (**P < 0.01; ns = nonsignificant). Data is represented by mean ± SEM. Experiment schematic was created in Biorender.com.

**Figure S4: Low molecular weight mucins reduce *S. aureus* USA100 survival over time.** USA100 growth (A) in SCFM (grey), HMW mucin (yellow), and LMW mucin (blue) at 24 hours. USA100 growth (B) and GFP-fluorescence (C) over time at 4-, 8-, 12-, and 24-hours in different SCFM conditions. Correlation (D) of USA100 growth and fluorescence at the 24-hour timepoint (R2= 0.9919). One-way ANOVA was performed on growth in LMW and HMW mucin (****P < 0.0001). (Two-way ANOVA was performed on USA300 CFUs over time (*P ≤ 0.05, **P < 0.01 ***P < 0.001). Data is represented by mean ± SEM.

**Figure S5: Low molecular weight mucins reduce *S. aureus* USA100 biofilm biomass.** Representative Z-stack images of USA100 (A) biofilm in SCFM with and without eDNA or LMW mucins after 24 hours. USA100 biomass (B) in each condition and average aggregate size in SCFM with LMW mucin with and without eDNA of 3-5 biological replicates and 5 images taken of each condition. One-way ANOVA was performed on volumetric measurement (***P* ≤ 0.01, *****P* ≤ 0.0001) and Welch’s t-test performed on aggregation area (ns = nonsignificant). Data is represented by mean ± SEM.

**Figure S6: Reducing low molecular weight mucin concentration partially restores *S. aureus* USA100 growth and aggregate size.** Representative z-stack images of USA100 (A) in SCFM with 2 mg/mL mucin (Left) or 5 mg/mL mucin (Right) after 24 hours at 40x. Scale bar represents 50 µm. Quantification of (C) volumetric and (D) aggregate size of 5 biological replications with 3-6 images taken of each well. CFUs of USA100 (D) after 24 hours of growth. One-way ANOVAs were performed on CFUs, and Welch’s t-tests were performed on volume and aggregate area (**P* ≤ 0.05, ***P* ≤ 0.01, ****P* ≤ 0.001, *****P* ≤ 0.0001). Data is represented by mean ± SEM.

**Figure S7: Autofluorescence of SCFM with and without eDNA or mucin.** Fluorescence baseline of uninfected conditions of SCFM, SCFM + eDNA, SCFM + LMW Mucin, and SCFM + eDNA + Mucin measured at 488/510 Excitation/Emission. Lines represent means of 3-6 biological replicates.

**Figure S8. LMW mucin polymers broadly impact *S. aureus* laboratory and clinical isolate maximum endpoint GFP fluorescence intensity.** 24-hour GFP read of *S. aureus* laboratory strains (A-F) and clinical isolates (G-O) in SCFM with no polymers (red), SCFM with eDNA (yellow), SCFM with LMW mucin (blue), and SCFM with eDNA and LMW mucin (purple). One-way ANOVAs were performed (*P ≤ 0.05,**P ≤ 0.01, ***P ≤ 0.001, ****P ≤ 0.0001, ns = not significant). Data is represented by mean ± SEM.

**Figure S9: Growth of laboratory or clinical isolates of *Staphylococcus aureus* in standard laboratory culture media with mucin.** Fluorescence baseline of uninfected conditions. (A) Laboratory and (B, C) clinical isolates (D, E) of *Staphylococcus aureus* grown in tryptic soy broth (TSB) with or without mucin with GFP fluorescence measured every 20 minutes over 24 hours. Lines represent means of 3-6 biological replicates.

**Table S1. Bacterial Strains**

| **Strain or Plasmid** | **Description** | **Reference** |
| --- | --- | --- |
| ***Plasmids*** | | |
| pCM29 | *S. aureus sarAP1* promoter | (1) |
| ***Staphylococcus aureus strains*** | | |
| *S. aureus* LAC 13c | USA300 CA-MRSA, erm^S^ | (2) |
| *S. aureus* USA100 | USA100 Japan Clone | (3) |
| *S. aureus* LAC 13c WT + GFP | USA300 CA-MRSA + pCM29 GFP, Cm^R^ | (4) |
| *S. aureus* USA100 + GFP | USA100 + pCM29 GFP, Cm^R^ | (3) |
| *S. aureus* Newman + GFP | Newman + pCM29 GFP, Cm^R^ | This work |
| *S. aureus* 502A + GFP | Strain 502A + pCM29 GFP, Cm^R^ | This work |
| *S. aureus* SH1000 + GFP | Strain SH1000 + pCM29 GFP, Cm^R^ | This work |
| *S. aureus* SA0831 + GFP | CF clinical Isolate + pCM29, Cm^R^ | This work |
| *S. aureus* CRS42 + GFP | Clinical Isolate CRS42 + pCM29 GFP, Cm^R^ | This work |
| *S. aureus* CRS48 + GFP | Clinical Isolate CRS48 + pCM29 GFP, Cm^R^ | This work |
| *S. aureus* CRS55 + GFP | Clinical Isolate CRS55 + pCM29 GFP, Cm^R^ | This work |
| *S. aureus* CRS56 + GFP | Clinical Isolate GP56 + pCM29 GFP, Cm^R^ | This work |
| *S. aureus* CRS59 + GFP | Clinical Isolate GP59 + pCM29 GFP, Cm^R^ | This work |
| *S. aureus* CRS24 + GFP | Clinical Isolate CRS24 + pCM29 GFP, Cm^R^ | This work |
| *S. aureus* CRS01 + GFP | Clinical Isolate GP01 + pCM29 GFP, Cm^R^ | This work |
| *S. aureus* CRS25 + GFP | Clinical Isolate GP25 + pCM29 GFP, Cm^R^ | This work |
| ***Staphylococcus epidermidis*** | | |
| *S. epidermidis* M23864:W2 | Human skin isolate | (5) |
| *S. epidermidis* M0881 | Human nasal isolate | (5) |
| ***Pseudomonas aeruginosa*** | | |
| *P. aeruginosa* PA01 | Common Pseudomonas laboratory strain | (6) |
| *P. aeruginosa* PAM57-15 | CF clinical isolate | (7) |
| ***Nontypeable Haemophilus Influenzae*** | | |
| NTHi 86-028NP | Minimally passaged clinical isolate | (8) |
| ***Streptococcus sanguinis*** | | |
| *S. sanguinis* | SK36 | (9) |

**SUPPLEMENTAL REFERENCES**

1. Pang YY, Schwartz J, Thoendel M, Ackermann LW, Horswill AR, Nauseef WM. 2010. agr-Dependent interactions of Staphylococcus aureus USA300 with human polymorphonuclear neutrophils. J Innate Immun 2:546-59.

2. Heim CE, Bosch ME, Yamada KJ, Aldrich AL, Chaudhari SS, Klinkebiel D, Gries CM, Alqarzaee AA, Li Y, Thomas VC, Seto E, Karpf AR, Kielian T. 2020. Lactate production by Staphylococcus aureus biofilm inhibits HDAC11 to reprogramme the host immune response during persistent infection. Nat Microbiol 5:1271-1284.

3. Kiedrowski MR, Gaston JR, Kocak BR, Coburn SL, Lee S, Pilewski JM, Myerburg MM, Bomberger JM. 2018. Staphylococcus aureus Biofilm Growth on Cystic Fibrosis Airway Epithelial Cells Is Enhanced during Respiratory Syncytial Virus Coinfection. mSphere 3.

4. Huffines JT, Boone RL, Kiedrowski MR. 2024. Temperature influences commensal-pathogen dynamics in a nasal epithelial cell co-culture model. mSphere 0:e00589-23.

5. Otto M. 2009. Staphylococcus epidermidis--the 'accidental' pathogen. Nat Rev Microbiol 7:555-67.

6. Moreau-Marquis S, O'Toole GA, Stanton BA. 2009. Tobramycin and FDA-approved iron chelators eliminate Pseudomonas aeruginosa biofilms on cystic fibrosis cells. Am J Respir Cell Mol Biol 41:305-13.

7. Henderson AG, Davis JM, Keith JD, Green ME, Oden AM, Rowe SM, Birket SE. 2022. Static mucus impairs bacterial clearance and allows chronic infection with Pseudomonas aeruginosa in the cystic fibrosis rat. Eur Respir J 60.

8. Mason KM, Raffel FK, Ray WC, Bakaletz LO. 2011. Heme utilization by nontypeable Haemophilus influenzae is essential and dependent on Sap transporter function. J Bacteriol 193:2527-35.

9. Scoffield JA, Wu H. 2015. Oral streptococci and nitrite-mediated interference of Pseudomonas aeruginosa. Infect Immun 83:101-7.
